# Alloying at a Subnanoscale Maximizes the Synergistic Effect on the Electrocatalytic Hydrogen Evolution

**DOI:** 10.1002/anie.202209675

**Published:** 2022-08-23

**Authors:** Quan Zou, Yuji Akada, Akiyoshi Kuzume, Masataka Yoshida, Takane Imaoka, Kimihisa Yamamoto

**Affiliations:** ^1^ Laboratory for Chemistry and Life Science (CLS) Institute of Innovative Research (IIR) Tokyo Institute of Technology 4259 Nagatsuta Midori-ku, Yokohama 226-8503 Japan; ^2^ JST-ERATO YamamotoAtom Hybrid Project Tokyo Institute of Technology 4259 Nagatsuta Midori-ku, Yokohama 226-8503 Japan

**Keywords:** Alloys, Catalysis, Subnanoparticles, Synergistic Effect

## Abstract

Bonding dissimilar elements to provide synergistic effects is an effective way to improve the performance of metal catalysts. However, as the properties become more dissimilar, achieving synergistic effects effectively becomes more difficult due to phase separation. Here we describe a comprehensive study on how subnanoscale alloying is always effective for inter‐elemental synergy. Thirty‐six combinations of both bimetallic subnanoparticles (SNPs) and nanoparticles (NPs) were studied systematically using atomic‐resolution imaging and catalyst benchmarking based on the hydrogen evolution reaction (HER). Results revealed that SNPs always produce greater synergistic effects than NPs, the greatest synergistic effect was found for the combination of Pt and Zr. The atomic‐scale miscibility and the associated modulation of electronic states at the subnanoscale were much different from those at the nanoscale, which was observed by annular‐dark‐field scanning transmission electron microscopy (ADF‐STEM) and X‐ray photoelectron spectroscopy (XPS), respectively.

## Graphical abstract

Phase separation in nanoparticles is undesirable from the viewpoint of the synergistic effects between elements in catalytic applications. Here, we propose the general concept that subnanoparticles are not phase‐separated and are an effective means for the expression of elemental synergies.

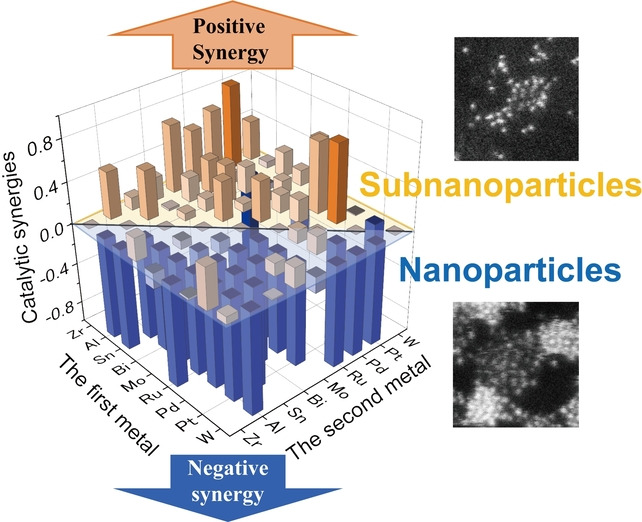

## Introduction

Alloying metals is one of the most common and effective ways to change material properties and strengthen metallic materials.[^1^, ^2^, ^3^] Alloying also is used to impart catalytic, magnetic and other properties.^[4]^ The concept of metal alloys has been applied to other materials, such as polymer alloys in organic macromolecules,[^5^, ^6^] ceramic alloys in metal oxides,[^7^, ^8^, ^9^] nitrides[^10^, ^11^, ^12^] and carbides.[^13^, ^14^] For all alloys, the two raw materials must have similar properties to avoid phase separation. In metallic alloys, combinations of atoms with similar electronegativity, atomic radius, and valence number generally form alloys more easily,[^15^, ^16^, ^17^] while polymers with similar polarity and hydrophilicity are preferred.[^18^, ^19^] If those similarities are not present, phase separation will occur, and the synergistic effect of alloying can be restricted to the interface of two phases.^[20]^ Therefore, atomic‐level compounds of dissimilar materials, such as metal‐ceramic alloys and metal‐polymer alloys (not metal complexes), do not exist.

However, these prerequisites are different for substances at the nanoscale and subnanoscale. Recent studies have revealed that alloys, not available in bulk, are available due to the improved miscibility at a nanoscale level.^[21]^ Furthermore, the miscibility limits are virtually eliminated at a subnanoscale level because no crystallization occurs,^[22]^ which distinguishes subnanoalloys^[23]^ from conventional alloys in bulk and nanoscale.^[24]^ For example, they exhibit outstanding catalytic properties,[^25^, ^26^, ^27^, ^28^] unique luminescence dependent on the number of atoms,^[29]^ and the emergence of super‐atomic properties[^30^, ^31^, ^32^]

The present study describes the fabrication of nano‐ and subnanoscale alloys with various combinations of elements of noble metal elements and base metal elements. An arc‐plasma deposition (APD) method allowed the preparation of various hybrid nanoparticles (NPs)[^33^, ^34^] and subnanoparticles (SNPs)[^35^, ^36^] with specific compositional ratios to systematically study the effects of constitution metals on both SNPs and NPs. The structures of these hybrid materials were elucidated using atomic‐resolution electron microscopy. In addition, the catalytic properties of the alloyed SNPs and NPs were evaluated using electrochemical HER, which is an ideal prototypical reaction that reflects the properties of constituent elements, and can elucidate fundamental characteristics of alloy substances. The synergistic effect of the various subnanoalloy and nanoalloy catalysts was examined based on these characterization data. The ability to create inter‐elemental hybrids of any metal or metal oxide with free and continuous composition ratios can expand the choice of nanomaterials.

## Results and Discussion

Optimization of the experimental conditions for APD in the preparation of uniform SNPs and NPs was conducted with respect to particle size. Figures S1 and S2 show the annular‐dark‐field scanning transmission electron microscopy (ADF‐STEM) images of Pt particles prepared using different applied voltages and pulse numbers under constant APD conditions (Table S1 Entry 1). Although every image showed highly dispersed Pt particles, the average particle size increased with the number of pulses. The borderline between Pt SNPs and NPs, where the average particle size was ca. 1 nm, was approximately ten pulses. Based on these results, six pulses and twenty pulses were selected to prepare SNPs and NPs, respectively. Note that in APD, the molar amount deposited per pulse differed depending on the target metal, even under the same experimental conditions (capacitance and voltage). Therefore, the optimal discharge voltages and capacitances were determined for each target metal (Table S1) and were used for all experiments so that the molar deposition rate per pulse measured by quartz crystal microbalance (QCM) would be equivalent among the elements. A variety of bimetallic Pt−M alloy SNPs and NPs (M: Pd, Ru, Mo, W, Al, Sn, Bi, and Zr) were prepared by alternately depositing Pt and M on the substrate using the experimental conditions shown in Table S1. Figures S3–S20 show the ADF‐STEM images of bimetallic SNPs and NPs, while Figures S21–S38 show the corresponding energy dispersive spectroscopy (EDS) mapping images of each SNP and NP alloy. In the present study, samples synthesized using alternative deposition with *x* pulses of element A and *y* pulses of element B were denoted as A_*x*_B_*y*_. For example, Pt_4_Zr_2_ represents the sample synthesized by 4 pulses of Pt and 2 pulses of Zr.

**Figure 1:**
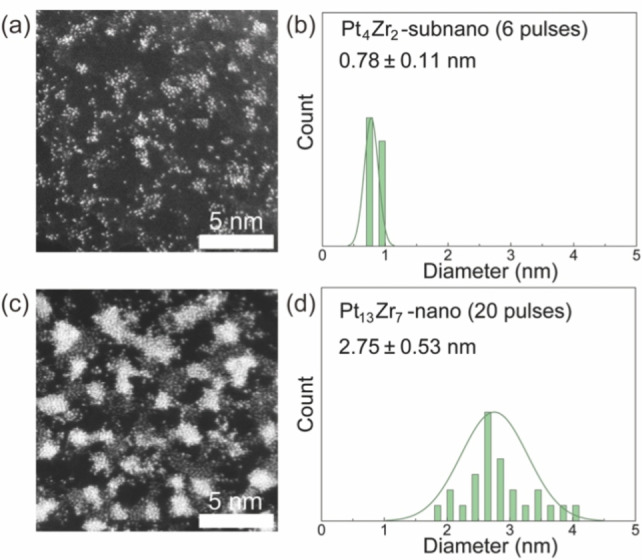
Characterization of PtZr alloy particles prepared from the different number of pulses. a,b) ADF‐STEM images and c,d) size distribution histograms. Panels (a) and (c) correspond to Pt_4_Zr_2_ SNPs, while panels (b) and (d) correspond to Pt_13_Zr_7_ NPs.

Figure 1 shows typical ADF‐STEM images of Pt_4_Zr_2_ SNPs and Pt_13_Zr_7_ NPs with size distribution histograms. The average diameters of SNPs and NPs were 0.78 and 2.75 nm, respectively. Apparently, the SNPs were less phase‐separated and more alloyed than the NPs according to atomic‐resolution ADF‐STEM images (Figure 1) and their time‐lapse movies (Movies 1–3). In principle, the brightness value of an atom in ADF‐STEM imaging is proportional to *Z* ^*a*^, where *Z* is the atomic number and *a* is a constant dependent on observation conditions (1<*a*<2).^[37]^ The Pt and Zr atoms were differentiated based on differences in brightness value. The NPs exhibited both high‐intensity and low‐intensity domains of about 3 nm, clearly indicating phase separation (Figure 1b). Most of the other Pt‐based bimetallic SNPs and NPs prepared by the APD method were similar to those of PtZr alloys as characterized by ADF‐STEM (Figures S3–S9) and STEM‐EDS mappings (Figures S21–S27). However, the PtW SNPs were an exception; its SNPs tended to aggregate to NPs with a diameter of about 2 nm (Figure S6).

**Figure 2:**
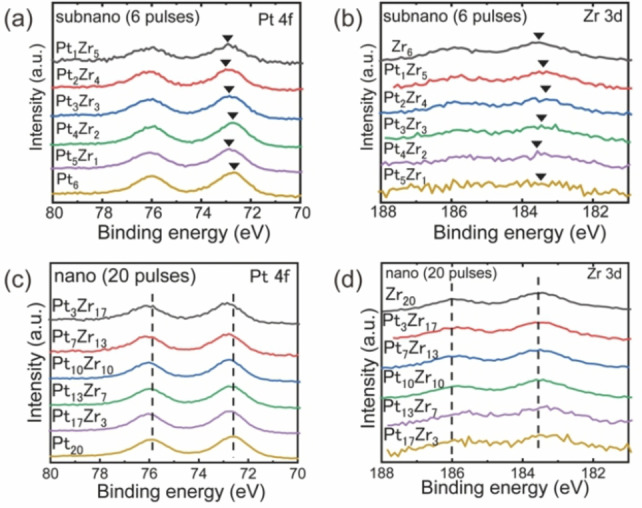
Comparison of electronic status between SNPs and NPs of different PtZr alloy compositions by X‐ray photoelectron spectroscopy (XPS). a) Pt 4*f* XPS of SNPs, b) Zr 3*d* XPS of SNPs, c) Pt 4*f* XPS of NPs, d) Zr 3*d* XPS of NPs

In general, the formation of heterometallic bonds affects the electronic state of the metal atoms through ligand effects. XPS were obtained to investigate the intermetallic effects (Figure S39). As depicted in Figure 2, the XPS of PtZr SNPs exhibited two distinct metallic Pt peaks that occurred at 72.8 and 75.8 eV, which correspond to Pt 4*f*_7/2_ and Pt 4*f*_5/2_, respectively. The multi‐peak fitting analysis of PtZr NPs (Figure S40) confirmed the existence of Pt^II^ peaks at 73.2 eV and 76.3 eV, suggesting that Pt atoms in both PtZr SNPs and PtZr NPs were partially oxidized. In addition, the Pt 4*f* region of PtZr SNPs displayed a slight positive shift compared with pure Pt_6_ SNP, indicating partial oxidation of Pt[^38^, ^39^] with the increase in Zr content. The Zr 3*d* peaks revealed that Zr exists as an oxide in a +4 valence state.^[40]^ The Pt 4*f* region of PtZr NPs also exhibited a positive shift compared with unary Pt_20_ NP. The other Pt‐based binary SNPs also exhibited similar positive shifts (Figure S39), indicating similar charge transfer effects for various combinations. Electronic state modulation through the formation of such heterometallic bonds can lead to a downshift of *d*‐band centers of Pt and influence their specificity in catalysis. In contrast to the gradual shifts of Pt 4*f* region in NPs, Pt 4*f* region of SNPs exhibited discrete shifts due to the subnano‐effect, which was also observed in previous reports.[^29^, ^41^, ^42^]

The enhanced miscibility at the subnanoscale level may increase synergy between dissimilar elements in the catalyst. Therefore, the catalytic activity of an electrochemical HER was determined to clarify the effect of subnano alloying on the catalyst function. The HER was used because of its simple mechanism; it reflects the properties of the elements in a straightforward manner and allowed the determination of specific alloying effects at the subnanoscale.

The HER performances were evaluated and compared systematically using Pt‐based SNPs and NPs prepared directly onto glassy carbon electrodes. The APD conditions for the preparation were the same as those for the preparation of samples for STEM observation. During the activity test, 10 cycles of electrochemical (EC) annealing were performed to remove the impurities and stabilize the SNPs. The activity of various samples was measured once only, to ensure that the activity we measured was the intrinsic activity of SNPs. To prevent the leaching of SNPs, the 0.05 M H_2_SO_4_ was selected as the electrolyte. Before the activity test, we compared the cyclic voltammetry (CV) curves of samples before and after 10 cycles of EC‐annealing, no changes of the CV of Pt SNPs were found (Figure S41), so we confirmed that the leaching and agglomeration of SNPs were avoided. Moreover, the period of every sample from synthesis to activity measurement was completed within twelve hours, combined with the STEM images, which were taken in 3 days after synthesis, we knew that agglomeration was successfully avoided for most samples, except combinations such as PtW SNPs.

To remove the possibility of dissolving and redeposition of Pt wire counter electrode, CV curves of a blank glassy carbon electrode before and after the HER activity test and linear sweep voltammogram (LSV) curves before and after 10 cycles of EC‐annealing were measured (Figures S42 and S43). No significant changes in CV and LSV curves were observed. Figure S44 shows the LSV polarization curves recorded in cathodic scans for Pt‐based binary alloys with various compositional metals at 0.05 M H_2_SO_4_. As a reference, LSV curves of various unary metal SNPs (Table S2 and S3) also were recorded in the same electrolyte. The sensitivity of the current response to applied overpotential is defined as the Tafel slope, which contains information about rate‐determining steps. As described previously, three major steps occur during hydrogen generation in acidic solution.[^16^, ^43^]

- Adsorption of H on the surface of catalytic sites, which is called the Volmer step: H^+^_(aq)_+e^−^→H_ads_
- The combination of adsorbed H and H^+^ to form H_2_, which is called the Heyrovsky step: H_ads_+H^+^_(aq)_+e^−^→H_2(g)._
- Formation of H_2_ directly from adsorbed H, which is called the Tafel step: 2 H_ads_→H_2(g)._

**Figure 3:**
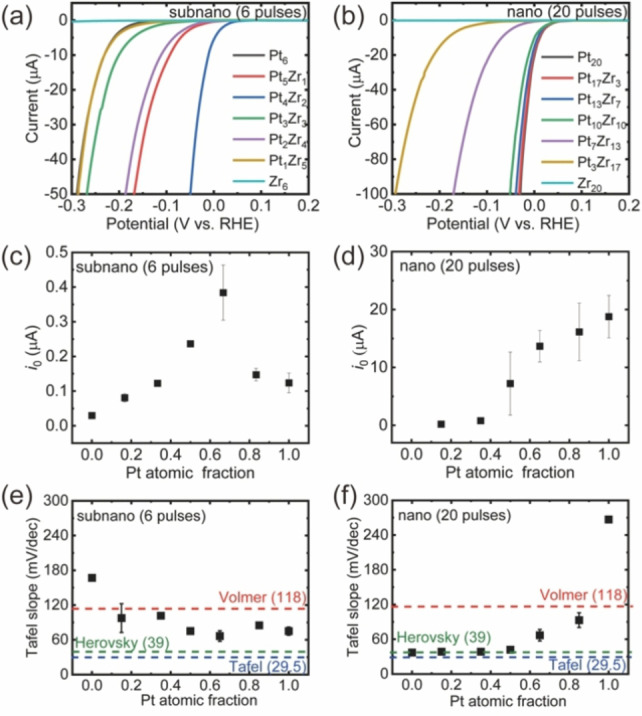
Comparison of electrocatalytic activity of SNPs and NPs of PtZr alloys for the hydrogen evolution reaction (HER). a,b) Original linear sweep voltammogram (LSV) curves of PtZr alloy SNPs and NPs in the aqueous argon‐saturated electrolyte (0.05 M H_2_SO_4_). c,d) Exchange currents (*i*_0_) of HER derived by the Tafel plots. e,f) The Tafel slope values of HER. Panels (a), (c), and (e) correspond to SNPs; panels (b), (c), and (f) correspond to NPs

Although elucidation of the exact reaction mechanism of different catalysts is generally difficult, the Tafel slope gives information about the rate‐determining step (RDS) of HER.^[44]^ In aqueous H_2_SO_4_ electrolytes, Tafel slopes are 118, 39, and 29.5 mV dec^−1^ when steps (a), (b) and (c) are the RDS, respectively. Tafel plots obtained from the polarization curves of the six samples are presented in Figure S44. The values of the Tafel slope for Pt_3_Mo_3_, Pt_3_W_3_, Pt_3_Bi_3_, Pt_3_Sn_3_, and Pt_3_Al_3_ SNPs were 116, 135, 112, 389, and 125 mV dec^−1^, respectively, indicating that the RDSs are the Volmer step (a) for these five SNPs. These results demonstrate that the adsorption of hydrogen atoms on these five SNPs was relatively weak and the surface coverage of hydrogen atoms on the catalyst surface was relatively low, even with the application of overpotential. In contrast, the Tafel slopes of Pt_6_ and Pt_3_Zr_3_ SNPs were 70 and 74 mV dec^−1^, respectively, which are greater than that for Pt NPs (37 mV dec^−1^) where the Heyrovsky or Tafel step is dominant, and less than those of the five types of alloy SNPs where the Volmer step is dominant. This result was attributed to the moderate adsorption energy of hydrogen on Pt_3_Zr_3_, which plays an important role in the excellent catalytic activity in Figure 3b. However, the determinants of the Tafel slope are complex and should be examined using a microkinetic model. Note that the surface structure of SNP catalysts is not well‐defined, thus this is only a rough indicator.

For comparison, the catalytic synergy of PtZr combination was investigated at both the subnanoscale and nanoscale levels. The PtZr binary SNPs exhibited greater HER performance compared to their unary Pt and Zr SNPs (Figure 3). Combination with the STEM results (Figure 1) revealed the PtZr SNPs were fully alloyed, indicating that enhanced performance was due to the alloying effects. In addition, as illustrated in Figure 3c, the exchange currents of these electrocatalysts (Pt_6_, Pt_5_Zr_1_, Pt_4_Zr_2_, Pt_3_Zr_3_, Pt_2_Zr_4_, Pt_1_Zr_5_, and Zr_6_) were 0.13, 0.15, 0,29, 0.25, 0,13, 0.08, and 0.03 μA, respectively.

Catalysts with a Pt to Zr compositional ratio of 4 to 2 (Pt_4_Zr_2_) exhibited the highest exchange current, indicating a maximum synergistic effect at that ratio for PtZr subnanoalloys (Pt to Zr). The exchange currents of PtZr NPs were nearly a linear average of Pt_20_ and Zr_20_ (Figure 3d), which indicated that a very weak synergistic effect occurred in PtZr NPs. In addition, the Tafel slope of PtZr NPs (Figure 3f) also increased with Zr content, revealing that Pt played a main role as reactive sites and Pt and Zr of PtZr NPs worked independently in HER. The Tafel slope of most PtZr NPs being similar to Pt confirmed that Pt plays the main role as a reactive site in PtZr NPs. To examine the universality of synergistic effects among bimetallic NPs, a series of bimetallic NPs were prepared with 20 pulses on APD, and the electrochemical catalytic activity was compared among Pt‐based alloy NPs with the same loading molar amount. No Pt‐based binary catalysts showed a higher exchange current than the Pt NPs, the Pt_10_Al_10_ NPs exhibited the worst performance, as shown in Table S2.

**Figure 4:**
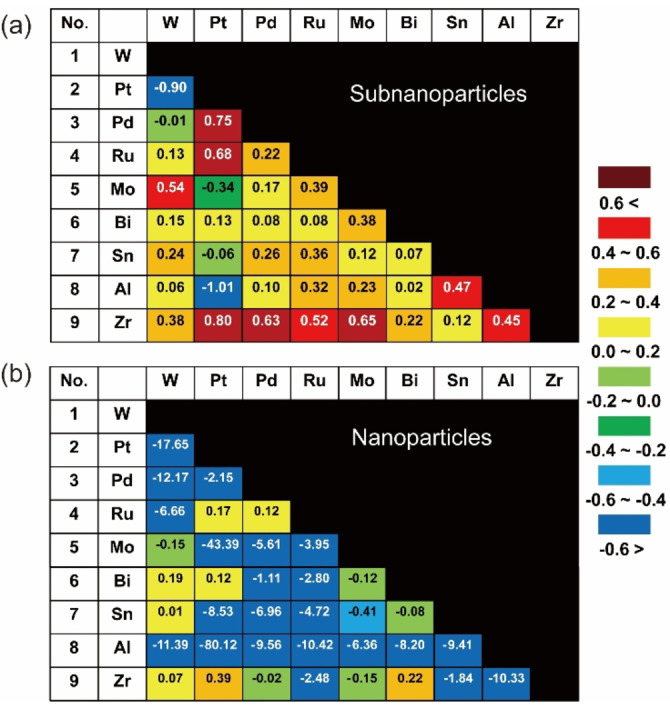
Synergistic effect indexes of various alloy particles for the electrocatalytic HER: a) SNP indexes; b) NP indexes

The reasons for the greater synergy with binary alloying of SNPs than with NPs and the better HER performance by PtZr binary SNPs compared to the other SNPs were investigated. A plausible explanation involves the significant modification of the electronic state of Pt upon alloying Pt and Zr due to ligand effects,^[45]^ which can weaken the adsorption of H_ads_ in the alloyed Pt. The strong adsorption of hydrogen to Pt atoms impeded the HER performance of Pt^[46]^ (Figure 4). Under this assumption, the reduced bonding strength of Pt−H_ads_ in the alloyed Pt atoms should facilitate the HER process according to the Sabatier principle. Thus, desorption of hydrogen should be the RDS in Pt SNPs, otherwise, PtZr SNPs would have decreased HER activity in comparison to Pt SNPs. The Tafel slopes for Pt_6_ and Pt_4_Zr_2_ which were 70 mV dec^−1^ and 74 mV dec^−1^, respectively (Volmer–Heyrovsky reaction mechanism), was consistent with the assumption that acceleration of the hydrogen desorption step in Pt_6_ and Pt_2_Zr_4_ enhanced HER activity (i.e., the best HER performance of PtZr SNPs is achieved by atomic‐level mixing of Zr and Pt and the balance of the charge transfer).

To clarify the positioning of the bimetallic SNPs composed of Pt and Zr, and to compare the synergistic effect modes of SNPs and NPs on HER performance, all possible relationships between HER activity and composition pair were analyzed. The comparative analysis among elements should be based on activity per surface area. However, the comparisons done here were based on activity per mole of atoms because the electrochemical surface area (ECSA) of SNP catalysts was not available. The number of moles of atoms deposited per discharge pulse was kept constant in the arc‐plasma deposition process. Therefore, the raw current value can represent the activity per mole of atoms for catalysts obtained with the same number of discharge pulses. The STEM observations (Figures. S3–S20) showed that no significant difference was present in particle size and particle morphology in bimetallic SNPs and NPs, respectively, composed of different elements. According to the observations, no significant difference existed in the specific surface area of SNPs with different compositions; therefore, the activity per mole of atoms could be used as an indicator.

To compare the difference in intrinsic properties between SNPs and NPs, an HER synergistic index (HSI) was proposed and defined by the following formula:

(1) $$HSI=\left(2i_{0}\left(M_{x}^{1}M_{y}^{2}\right)-i_{0}\left(M_{x}^{1}\right)-i_{0}\left(M_{y}^{2}\right)\right)/2i_{0}\left(M_{x}^{1}M_{y}^{2}\right)$$

where *i*_0_(M^1^), *i*_0_(M^2^), and *i*_0_($M_{x}^{1}M_{y}^{2}$ ) represent the HER exchange current of single component M^1^, single component M^2^, and bimetallic M^1^_*x*_M^2^_*y*_ (*x* and *y* represent concentrations of M^1^ and M^2^, respectively). Based on the HSI, bimetal combinations were systematically investigated to create two diagrams of bimetallic SNPs and NPs (Figure S45).

The diagram of SNPs in Figure 4a shows the HSI values for all bimetallic SNP and NP combinations (Tables S2 and S3). All of the Zr‐based SNPs had a positive HSI, indicating that Zr played an important role in facilitating HER performance; the best HSI was found in PtZr, revealing that the optimal value was reached in the combination of PtZr. Conversely, viewing the diagram based on Pt suggested the HER activities dependent on the additive metals. The HSI values were in the following order: PtZr>PtBi>PtSn>PtMo>PtW>PtAl. The positive shifts of the Pt 4*f* binding energies in XPS for all Pt‐based subnanoalloys (Figure S39) demonstrated the homogeneous alloying of metal and metal oxides in SNPs. There are two exceptions (PtW and PtAl) in which significant negative HSI values were found for Pt‐based SNPs (Figure 4a). The negative HSIs were attributed to either aggregation or the intrinsic properties of the formed metal oxides^[47]^ (Figures S6 and S7).

In contrast, the diagram of NPs in Figure 4b shows that many bimetallic NPs exhibited negative HSIs. Some of which seem to contradict the results of previous studies. For example, a previous study^[48]^ reported that a combination of Pd and Ru in PdRu NPs can lead to several times greater HER activity than unary Pd and Ru, which is inconsistent with the present results. However, the enhanced HER performance of this previous study was due mainly to the core–shell structure, which prevented unfavorable phase segregation. Similar results for other combinations, such as AuNi,^[49]^ AuIr,^[50]^ CuNi,^[51]^ and others,^[52]^ can be found in previous reports. These previous results demonstrating positive synergistic effects for HER are ensemble results of the morphology,^[53]^ facets,^[54]^ supports,^[55]^ or size effects,^[56]^ where the intrinsic synergistic effect of bimetallic NPs was dwarfed.[^57^, ^58^, ^59^, ^60^]

In general, NPs composed of dissimilar elements easily phase‐separated into Janus‐type NPs or core–shell NPs. In Janus‐type NPs,^[61]^ the hetero‐interface area is very small relative to the entire volume of the particle. In core–shell phase‐separated NPs,^[62]^ the hetero‐interface is not exposed on the surface. In many cases, it is necessary to create appropriate structures for the applications by engineering the synthetic method[^63^, ^64^] to take advantage of the synergistic effect of NPs. The degree of utilizing mixed phases determined the degree of synergistic effect in catalytic performance. On the other side, the structure of SNPs is homogeneous and fluidic, as explained later, and even elements that favor the phase separation can exist as a mixed phase in the atomic‐level short‐range order. Thus, the activity can be maximized by downsizing bimetallic catalysts to SNPs.

A comparison of the two diagrams in Figure 4 reveals that SNPs are a series of promising candidates for catalysis, because the bimetallic SNPs generally possessed higher HSI values than their corresponding bimetallic NPs. The high HSI between elements with large differences in the electronegativity, such as Pt and Zr, is a characteristic of SNPs that is not found in NPs.

A unique feature of SNPs that differentiates them from NPs is their metastable nature. Atoms in SNPs move continuously during electron microscopy observation and do not form stable phase‐separated domains. To understand the state of mixing, the X‐Y coordinate of every Pt and Zr atom was extracted from the atomic‐resolution ADF‐STEM movies and the numbers of Pt−Pt, Zr−Zr, and Pt−Zr bonds were counted to obtain statistics. The different atoms were marked with different colors and their movements were tracked through automated image processing (Movies 1 and 2). By calculating the distances between different atoms, the presence of chemical bonds was determined. For PtZr SNPs as an example, the distance between the Pt and Zr atoms changed with time. The process of counting the different bonds was performed by a program that extracts the coordinates and brightness of each observed atom and identifies the element with a threshold value (Movie 3).

**Figure 5:**
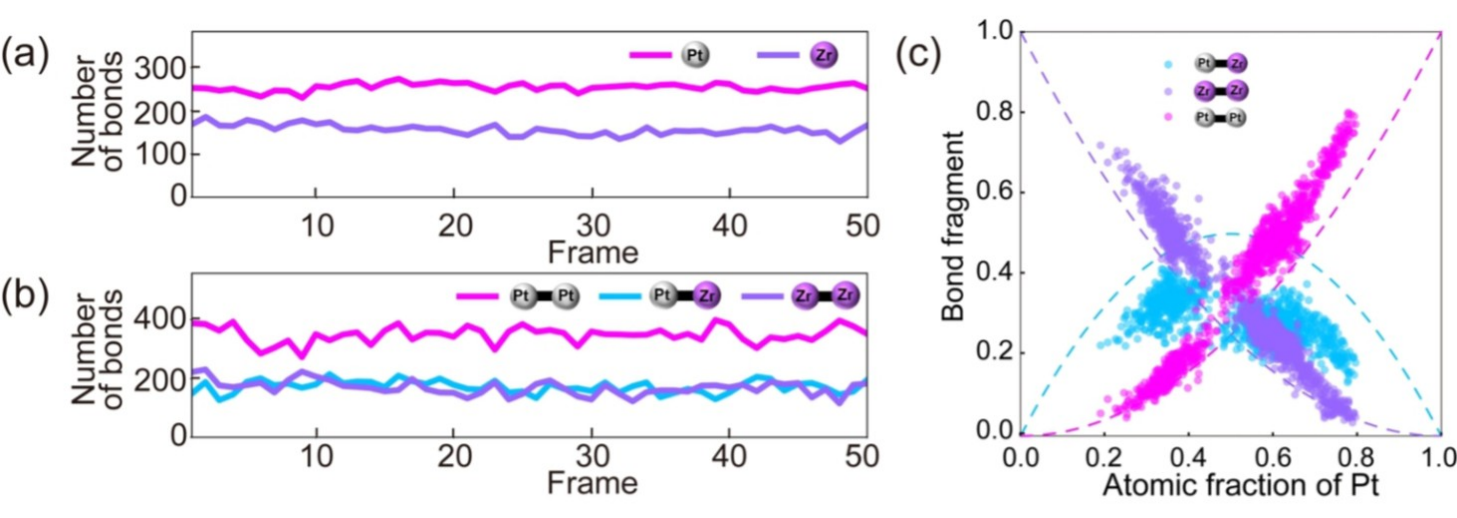
Bond analysis of an ADF‐STEM movie clip of Pt_4_Zr_2_ SNPs (Movies 1–3). a) The local numbers of Pt and Zr atoms found in each frame. b) The local numbers of Pt−Pt, Pt−Zr, and Zr−Zr bonds found in each frame. c) Relationship between the local molar fraction of Pt and the fractions of Pt−Pt, Pt−Zr, and Zr−Zr bonds. Dotted lines show the proportion of each bond that occurs statistically, assuming no enthalpy difference in bond formation between the same and different species

The existence of a Pt−Zr bond was defined as having a distance smaller than 0.35 nm, based on the results of a pair distribution function (Figure S46). If the Pt and Zr atoms were mixed randomly without any short‐range order, the ratio of Pt−Zr : Pt−Pt : Zr−Zr should be equal to the ratio of N(Pt)^2^ : 2 N(Pt)N(Zr) : N(Zr)^2^, in which N(Pt) and N(Zr) represent the number of Pt and Zr atoms, respectively. For a Pt concentration of 67 % (indicated by the dotted line in Figure 5c), the percentages of Pt−Pt, Pt−Zr, and Zr−Zr should be approximately 44 %, 44 %, and 11 % (4 : 4 : 1) in a random system. However, the results of the present observations showed that the percentage of Pt−Zr remained stable at about 25 %, with slight fluctuations over time (Figure 5b). In the subnanometer system, Pt and Zr atoms were close to a “regular solution” where they did not exhibit any significant attraction or repulsion with respect to each other. Moreover, an examination of the relationship between Pt concentration and bond percentage revealed that an increase in Pt concentration resulted in an initial increase in the percentage of Pt−Zr followed by a decrease (Figure 5c), with the greatest percentage of Pt−Zr of about 50 %. Compared to NPs, which were phase‐separated at the domain level, SNPs were still able to form heterometallic bonds. Thus, the spontaneous formation of such heterometallic bonds is likely to be the reason for the synergistic effect in SNPs that is not found in NPs.

The electrochemical study demonstrated that a composition of Pt_4_Zr_2_ was the best catalyst for HER. To provide insight into this result, DFT calculations were performed for simple Pt_*x*_Zr_*y*_ cluster models. The most stable cluster structures for each metal were explored and the results are shown in Table S4. The hydrogen adsorption energy (*E*_ads_) is defined as follows:

(2) $$E_{ads}=E\left(SNP+H\right)-E\left(SNP\right)+\frac{1}{2}E\left(H_{2}\right)$$

where *E*(SNP) and *E*(H_2_) are the energy of single SNP and H_2_ molecules, respectively. The expression *E*(SNP+H) represents the energy of a single SNP absorbed with one hydrogen atom. Table S4 shows that Zr atoms tend to form bonds with Pt atoms. In addition, the most stable structure of SNPs contained less than 5 Pt atoms, and Pt−Pt bonds were unavailable. With an increase in the number of Pt atoms, adsorption of hydrogen atoms gradually changed from Zr to Pt atoms. The Pt and Zr atoms which acted as adsorption hydrogen sites depended on which atoms were the majority in Pt_*x*_Zr_*y*_ SNPs.

The *E*_ads_ plotted against Pt atom composition ratio is shown in Figure S47. The SNP with the lowest hydrogen adsorption energy in a negative direction was Pt_4_Zr_2_ SNPs, which indicates that Pt_4_Zr_2_ had the weakest interaction with hydrogen among all of the Pt_*x*_Zr_*y*_ SNPs. According to the Sabatier principle, catalysts that experience a very strong interaction with hydrogen atoms are not good catalysts for HER, because the adsorbed hydrogen atoms minimally desorb as hydrogen molecules. Therefore, this calculational result can explain qualitatively why the greatest HER activity was observed for Pt_4_Zr_2_ SNPs.

## Conclusion

A comprehensive study of alloys on a nano‐ and subnanoscale consisting of various combinations of elements revealed that PtZr SNPs possess the greatest inter‐elemental synergy. They also exhibited the greatest activity with a composition of Pt_4_Zr_2_ (67 % atomic percentage of Pt). In this case, Pt was the zero‐valent metal, while Zr^0^ was oxidized Zr^IV^. Surprisingly, the greatest synergistic effect was observed for a mixture of metal and metal oxide atoms that are generally immiscible.

Overall, SNPs and NPs exhibited contrasting atomic arrangements. To clarify the arrangement of the atoms, atomic‐resolution ADF‐STEM images were acquired, and the atomic‐scale mixing was evaluated statistically. The Pt−Zr bonds in SNPs account for 25 % of the total bonds, which indicates 55 % of the homogeneous mixture of Pt and Zr in a regular solution. In contrast, Pt and Zr atoms are segregated in NPs, meaning SNPs are much more miscible than NPs, suggesting more feasible inter‐elemental synergies in the subnanoscale. These results indicated that nanoalloys and subnanoalloys belong to completely different categories of materials.

Recent studies on trimetallic subnanoalloys such as AuAgCu SNPs^[65]^ and PdAuCu SNPs^[25]^ for catalysis demonstrated the enhanced miscibility and the corresponding synergy in catalysis also available in multimetallic systems. The present conclusion may also be applicable to multi‐metallic SNPs which are a very attractive and unexplored group of materials for catalysts and other applications.

## Conflict of interest

The authors declare no conflict of interest.

1

## Supporting information

As a service to our authors and readers, this journal provides supporting information supplied by the authors. Such materials are peer reviewed and may be re‐organized for online delivery, but are not copy‐edited or typeset. Technical support issues arising from supporting information (other than missing files) should be addressed to the authors.

Supporting Information

Supporting Information

Supporting Information

Supporting Information

## Data Availability

The data that support the findings of this study are available from the corresponding author upon reasonable request.
