# Supplementary material for: Alloying at a Subnanoscale Maximizes the Synergistic Effect on the Electrocatalytic Hydrogen Evolution
Source: Angew Chem Int Ed Engl. 2022 Aug 23;61(40):e202209675. doi: 10.1002/anie.202209675 (PMC9804976; doi:10.1002/anie.202209675)
Supplement: Supplementary file 1 — Supporting Information [file ANIE-61-0-s001.pdf]

Angewandte Chemie
Eine Zeitschrift der Gesellschaft Deutscher Chemiker

Supporting Information

Alloying at a Subnanoscale Maximizes the Synergistic Effect on the Electrocatalytic Hydrogen Evolution

Q. Zou, Y. Akada, A. Kuzume, M. Yoshida, T. Imaoka, K. Yamamoto**

Table of Contents

Section S1. Experimental section
S1.1 Chemicals
S1.2 Preparation of SNPs and NPs
S1.3 Characterization of SNPs and NPs
S1.4 Electrochemical measurements for the hydrogen evolution reaction (HER)
S1.5 Processing of ADF-STEM images

Section S2. APD conditions for preparation of SNPs and NPs
Table S1. APD deposition conditions

Section S3. STEM images and elemental mapping of bimetallic SNPs and NPs
Figure S1. ADF-STEM and particle size variations of Pt with different applied voltages.
Figure S2. ADF-STEM and particle size variations of Pt with different pulse numbers.
Figure S3. ADF-STEM and particle size histograms of Pt_3Pd_3 SNPs and $Pt_{10}Pd_{10}$ NPs.
Figure S4. ADF-STEM and particle size histograms of Pt_3Ru_3 SNPs and $Pt_{10}Ru_{10}$ NPs.
Figure S5. ADF-STEM and particle size histograms of Pt_3Mo_3 SNPs and $Pt_{10}Mo_{10}$ NPs.
Figure S6. ADF-STEM and particle size histograms of Pt_3W_3 SNPs and $Pt_{10}W_{10}$ NPs.
Figure S7. ADF-STEM and particle size histograms of Pt_3Al_3 SNPs and $Pt_{10}Al_{10}$ NPs.
Figure S8. ADF-STEM and particle size histograms of Pt_3Sn_3 SNPs and $Pt_{10}Sn_{10}$ NPs.
Figure S9. ADF-STEM and particle size histograms of Pt_3Bi_3 SNPs and $Pt_{10}Bi_{10}$ NPs.
Figure S10. ADF-STEM and particle size histograms of Pd_3Ru_3 SNPs and $Pd_{10}Ru_{10}$ NPs.
Figure S11.ADF-STEM and particle size histograms of Pd_3Mo_3 SNPs and $Pd_{10}Mo_{10}$ NPs.
Figure S12. ADF-STEM and particle size histograms of Pd_3W_3 SNPs and $Pd_{10}W_{10}$ NPs.
Figure S13. ADF-STEM and particle size histograms of Pd_3Sn_3 SNPs and $Pd_{10}Sn_{10}$ NPs.
Figure S14. ADF-STEM and particle size histograms of Pd_3Bi_3 SNPs and $Pd_{10}Bi_{10}$ NPs.
Figure S15. ADF-STEM and particle size histograms of Pd_3Zr_3 SNPs and $Pd_{10}Zr_{10}$ NPs.
Figure S16. ADF-STEM and particle size histograms of Ru_3Mo_3 SNPs and $Ru_{10}Mo_{10}$ NPs.
Figure S17. ADF-STEM and particle size histograms of Ru_3W_3 SNPs and $Ru_{10}W_{10}$ NPs.
Figure S18. ADF-STEM and particle size histograms of Ru_3Sn_3 SNPs and $Ru_{10}Sn_{10}$ NPs.
Figure S19. ADF-STEM and particle size histograms of Ru_3Bi_3 SNPs and $Ru_{10}Bi_{10}$ NPs.
Figure S20. ADF-STEM and particle size histograms of Ru_3Zr_3 SNPs and $Ru_{10}Zr_{10}$ NPs.
Figure S21. Elemental mapping of Pt_3Pd_3 SNPs and $Pt_{10}Pd_{10}$ NPs.
Figure S22. Elemental mapping of Pt_3Ru_3 SNPs and $Pt_{10}Ru_{10}$ NPs.
Figure S23. Elemental mapping of Pt_3Mo_3 SNPs and $Pt_{10}Mo_{10}$ NPs.
Figure S24. Elemental mapping of Pt_3W_3 SNPs and $Pt_{10}W_{10}$ NPs.
Figure S25. Elemental mapping of Pt_3Al_3 SNPs and $Pt_{10}Al_{10}$ NPs.
Figure S26. Elemental mapping of Pt_3Sn_3 SNPs and $Pt_{10}Sn_{10}$ NPs.
Figure S27. Elemental mapping of Pt_3Bi_3 SNPs and $Pt_{10}Bi_{10}$ NPs.
Figure S28. Elemental mapping of Pd_3Ru_3 SNPs and $Pd_{10}Ru_{10}$ NPs.
Figure S29. Elemental mapping of Pd_3Mo_3 SNPs and $Pd_{10}Mo_{10}$ NPs.
Figure S30. Elemental mapping of Pd_3W_3 SNPs and $Pd_{10}W_{10}$ NPs.
Figure S31. Elemental mapping of Pd_3Sn_3 SNPs and $Pd_{10}Sn_{10}$ NPs.
Figure S32. Elemental mapping of Pd_3Bi_3 SNPs and $Pd_{10}Bi_{10}$ NPs.
Figure S33 Elemental mapping of Pd_3Zr_3 SNPs and $Pd_{10}Zr_{10}$ NPs.
Figure S34. Elemental mapping of Ru_3Mo_3 SNPs and $Ru_{10}Mo_{10}$ NPs.
Figure S35. Elemental mapping of Ru_3W_3 SNPs and $Ru_{10}W_{10}$ NPs.
Figure S36. Elemental mapping of Ru_3Sn_3 SNPs and $Ru_{10}Sn_{10}$ NPs.
Figure S37. Elemental mapping of Ru_3Bi_3 SNPs and $Ru_{10}Bi_{10}$ NPs.
Figure S38. Elemental mapping of Ru_3Zr_3 SNPs and $Ru_{10}Zr_{10}$ NPs

Section S4. X-ray photoelectron spectroscopy (XPS)
Figure S39. XPS of Pt 4*f* of Pt-based bimetallic (a) SNPs and (b) NPs.

Figure S40. Pt 4*f* and Zr 3*d* XPS of Pt_4Zr_2 (a, b) SNPs and (c, d) $Pt_{13}Zr_7$ NPs.

Section S5. Electrochemical measurements

Figure S41. Cyclic voltammetry (CV) curves of Pt SNPs and NPs before and after EC annealing.

Figure S42. CV curves of blank sample before and after HER activity test.

Figure S43. LSV curves of blank sample, blank sample after EC annealing and Pt_6 SNPs.

Table S2. Exchange current and Tafel slope of unary and bimetallic NPs.

Table S3. Exchange current and Tafel slope of unary and bimetallic SNPs.

Figure S44. (a, b) LSV recorded on cathodic scans and (c, d) Tafel slope of Pt-based bimetallic SNPs and NPs in 0.05 M H_2SO_4.

Section S6. HSI (HER synergistic effect index)

Figure S45. Relationship between HER activity and compositional ratio showing a (a) positive synergistic effect, (b) no synergistic effect, (c) negative synergistic effect.

Section S7. Determination of bond length

Figure S46. (a) Treatment procedures for ADF-STEM images, (b) subtraction of background and background thresholds for different atoms; (c) pair distribution functions for Pt-Pt, Pt-Zr, and Zr-Zr bonds.

Section S8. DFT calculations

Table S4. Most stable cluster structures and the lowest H adsorption energies (E_{ads}) against composition ratio. Green, gray, and pink spheres indicate Zr, Pt, and H atoms, respectively. The threshold value for bond length between metal atoms is 3 Å.

Figure S47. Effect of the number of Pt atoms in the SNP on E_{ads}. In each case, total number of atoms in one SNP was fixed to six, and the number of Zr atoms is given as the difference between six and the number of Pt atoms.

Section S1. Experimental Procedures

S1.1 Chemicals.

The pretreatment of graphene nanoplatelets was conducted as reported previously[1]. The graphene nanoplatelets purchased from Aldrich were washed with aq. HCl (300 mL, 3 mol mL^{-1}), CH_3OH, and purified water in sequence to remove the impurities. The CH_3OH in this study was purchased from Kanto Chemical Co., Inc. and the H_2SO_4 was purchased from Fujifilm Wako Pure Chemical Co., and both were used as received.

S1.2 Preparation of SNPs and NPs

The SNPs or NPs were prepared on graphene, a silicon plate, or a glassy carbon electrode (GCE) using a vacuum deposition method with a pulsed arc plasma source (Advance Riko, APS-1) equipped with metal cylinder targets. The arc pulse was generated with a frequency of 1 Hz with a period of 500 ms and current amplitude of 2 kA. The deposition amount of each metal element was determined by the number of pulses based on the amount of deposition calculated by the quartz crystal microbalance (QCM) method. Discharge voltage and capacity were adjusted according to the respective targets, as shown in Table S1.

S1.3. Characterization of SNPs and NPs

Annular dark-field scanning transmission electron microscopy (ADF-STEM) images were obtained using an aberration-corrected transmission electron microscope (Jeol, JEM-ARM200F) operated at an 80 kV acceleration voltage. The inner and outer collection angles used in recording the ADF-STEM images were 57 and 226 mrad, respectively. Probe current was fixed at 26 pA. The SNPs and NPs were processed directly onto graphene nanoplatelets using arc-plasma deposition (APD). The graphene nanoplatelets were deposited on thin holey carbon-film-coated Cu grids (Nissin EM Co., Ltd.) by drop-casting of the homogeneous suspension in methanol, followed by vacuum drying overnight.

X-ray photoelectron spectroscopy (XPS) was conducted with a Shimadzu ESCA-3400HSE instrument with Mg Kα X-ray (10 kV, 20 mA). The silicon substrates were cleaned in H_2SO_4 with ultrasonication for 15 minutes, rinsed with ultrapure water three times, and vacuum dried overnight. Various SNPs and NPs for XPS analysis were deposited on silicon substrates under the APD conditions shown in Table S1.

S1.4. Electrochemical measurements for the hydrogen evolution reaction (HER)

Electrochemical measurements were obtained using an electrochemical analyzer (ALS750A, CH Instruments) in standard three-electrode configuration. A platinum wire and reversible hydrogen electrode (RHE) were used as a counter electrode and reference electrode, respectively. The working electrode was a catalyst-modified glassy carbon disk electrode (GCE, 3.0 mm diameter). The SNPs and NPs were deposited directly on the GCE. Before deposition, the GCE was polished with 1-, 0.3-, and 0.005-μm alumina paste. The APD-processed electrode was used only once for electrochemical measurements. A typical HER measurement was conducted in 0.05 M H_2SO_4 electrolyte after argon bubbling for 15 min. Electrochemical cleaning of the catalyst surface deposited on the working electrode was conducted by scanning the working electrode potential from 0.06 to 1.00 V *vs*. RHE with a scan rate of 0.1 V s^{-1} for 21 segments. Next, cyclic voltammetry (CV) was conducted from 0.06 to 1.00 V at a scan rate of 50 mV s^{-1} for five segments. Finally, a Tafel plot was obtained from −0.3 to 0.2 V *vs*. RHE at a scan rate of 2 mV s^{-1}. The polarization curves were expressed as overpotential (η) *vs*. log current [log(i)] to create the Tafel plots[2]. By fitting the linear portion of the Tafel plots to the Tafel equation [$\eta = A \log(i/i_0)$], the Tafel slope (A) was obtained. The exchange current is i_0, which represents the intrinsic HER activity of catalysts under reversible conditions. The value of i_0 for the catalyst was obtained by extrapolating the zero tangent line to 0.0 V *vs*. RHE, which is the zero overpotential of HER. The resistance of our experimental setup (0.05 M H_2SO_4 solution) was 67 Ω. Therefore, the IR drop in the current range observed in our experiments (<100μA) was less than 7mV, which was within the experimental error. (Fig. S43)

S1.5. Processing of ADF-STEM images

All successive 50 frames in the ADF-STEM movie (Movie 1) were processed by following four steps using Image J software[3,4]. First, the original images acquired from STEM were upconverted from to 512 × 512 pixels to 1024 × 1024 pixels using the resize method (interpolation=Bicubic). Second, the background of resized images was subtracted using the Subtract Background method (radius=50). Third, the Gaussian Blur method (sigma=4) was applied to remove high-frequency noise from the images. Fourth, the x-y coordinates and the intensity values for local brightness maxima were searched by applying the Find Maxima method (prominence=0) to extract the positions and elements of atoms. The brightness intensity value was used to identify the element (Pt or Zr) in the images. The spots with an intensity lower than 3800 were removed from the list because they were considered background noise. The intensity histogram showed two maxima at 5050 and 9210 (Fig. S46), which correspond to Zr and Pt atoms, respectively. Therefore, the intermediate intensity was set as the threshold for elemental identification, and those with an intensity higher than the threshold were assigned to Pt, while those with a lower intensity were assigned as Zr. Extraction of chemical bonds from the list of atomic coordination values were conducted from all atomic pairs in the same frame. The threshold distance of bonds d_{th} was set to 0.35 nm. The movie visualizing atom mapping (colored circles) and chemical bonds (white line) (Movie 2) was made based on the extracted coordinates (x, y) of element and chemical bonds. The overlayed movie (Movie 3) of Movies 1 and 2 represents validation of the analysis.

Here, the numbers of atoms ($\boldsymbol{A_{Pt}}$, $\boldsymbol{A_{Zr}}$) and bonds ($\boldsymbol{B_{Pt-Pt}}$, $\boldsymbol{B_{Pt-Zr}}$, $\boldsymbol{B_{Zr-Zr}}$) for each frame were counted and the molar fraction of Pt in a field of view was calculated according to eq. S1. The ratio of each bond to each frame was calculated using the number of bonds (eqs. S2–S4). Figure 5(c) was acquired by plotting the relationship of $\boldsymbol{R(B_{Pt-Pt})}$ *vs*. $\boldsymbol{F(A_{Pt})}$, $\boldsymbol{R(B_{Pt-Zr})}$ *vs*. $\boldsymbol{F(A_{Pt})}$ and $\boldsymbol{R(B_{Zr-Zr})}$ *vs*. $\boldsymbol{F(A_{Pt})}$.

$$\boldsymbol{F(A_{Pt}) = \frac{A_{Pt}}{A_{Pt}+A_{Zr}}} \quad (\textbf{for each frame}) \qquad \text{(S1)}$$

$$\boldsymbol{R(B_{Pt-Pt}) = \frac{B_{Pt-Pt}}{B_{Pt-Pt}+B_{Pt-Zr}+B_{Zr-Zr}}} \qquad \text{(S2)}$$

$$\boldsymbol{R}(\boldsymbol{B}_{\boldsymbol{Pt-Pt}}) = \frac{\boldsymbol{B}_{\boldsymbol{Pt-Zr}}}{\boldsymbol{B}_{\boldsymbol{Pt-Pt}}+\boldsymbol{B}_{\boldsymbol{Pt-Zr}}+\boldsymbol{B}_{\boldsymbol{Zr-Zr}}} \quad \text{(S3)}$$

$$\boldsymbol{R}(\boldsymbol{B}_{\boldsymbol{Pt-Pt}}) = \frac{\boldsymbol{B}_{\boldsymbol{Zr-Zr}}}{\boldsymbol{B}_{\boldsymbol{Pt-Pt}}+\boldsymbol{B}_{\boldsymbol{Pt-Zr}}+\boldsymbol{B}_{\boldsymbol{Zr-Zr}}} \quad \text{(S4)}$$

Section S2. APD conditions for preparation of SNPs and NPs

Table S1. APD deposition conditions

Entry	Target elements	Capacitance (μF)	Voltage (V)	Loading mass (μg/pulse)
1	Pt	1080	70	0.051
2	Pd	1080	70	0.027
3	Ru	1080	100	0.026
4	Zr	1080	100	0.024
5	Al	360	70	0.007
6	Sn	360	80	0.031
7	Mo	1080	100	0.013
8	Bi	360	70	0.055
9	W	1080	150	0.048

Section S3. STEM images and elemental mapping of bimetallic SNPs and NPs

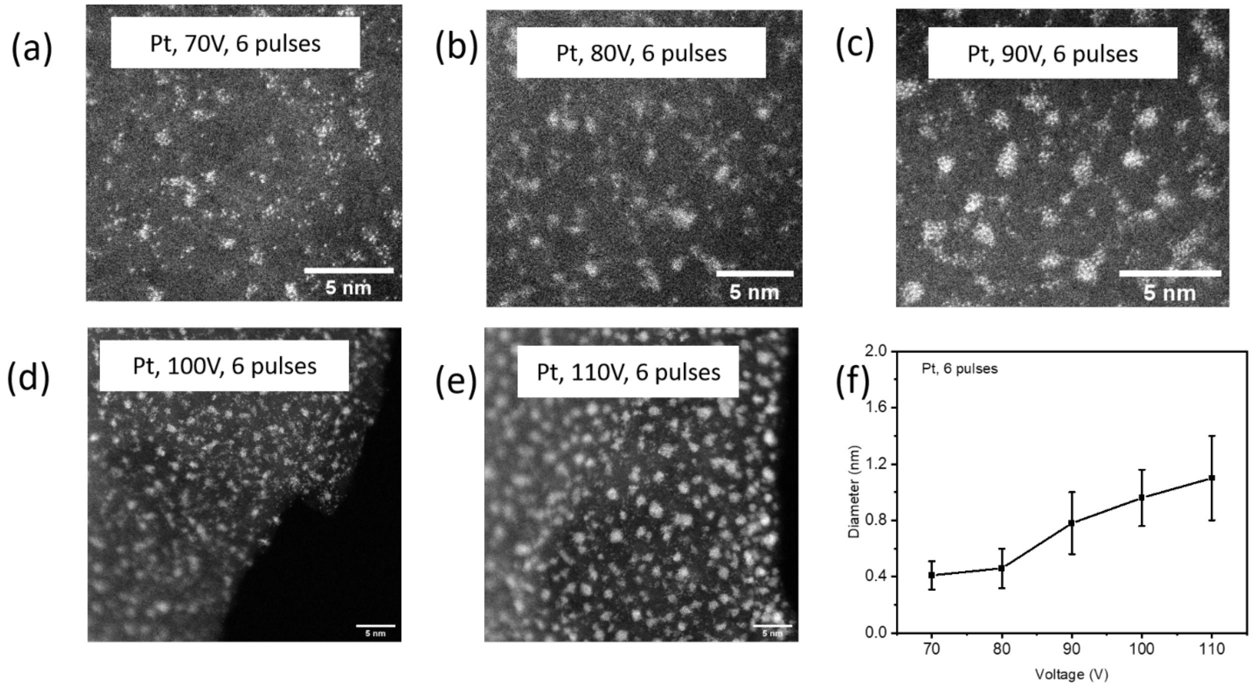

Figure S1. ADF-STEM and particle size variations in Pt with different applied voltages.

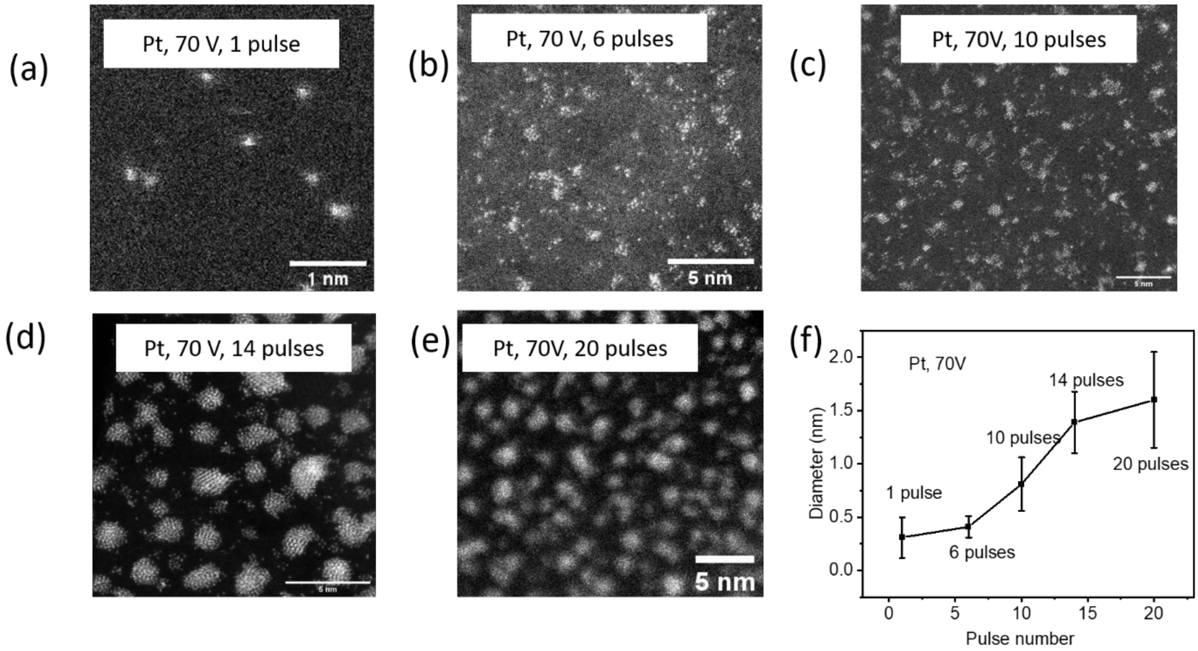

Figure S2. ADF-STEM and particle size variation of Pt with different pulse numbers at the applied voltage of 70 V.

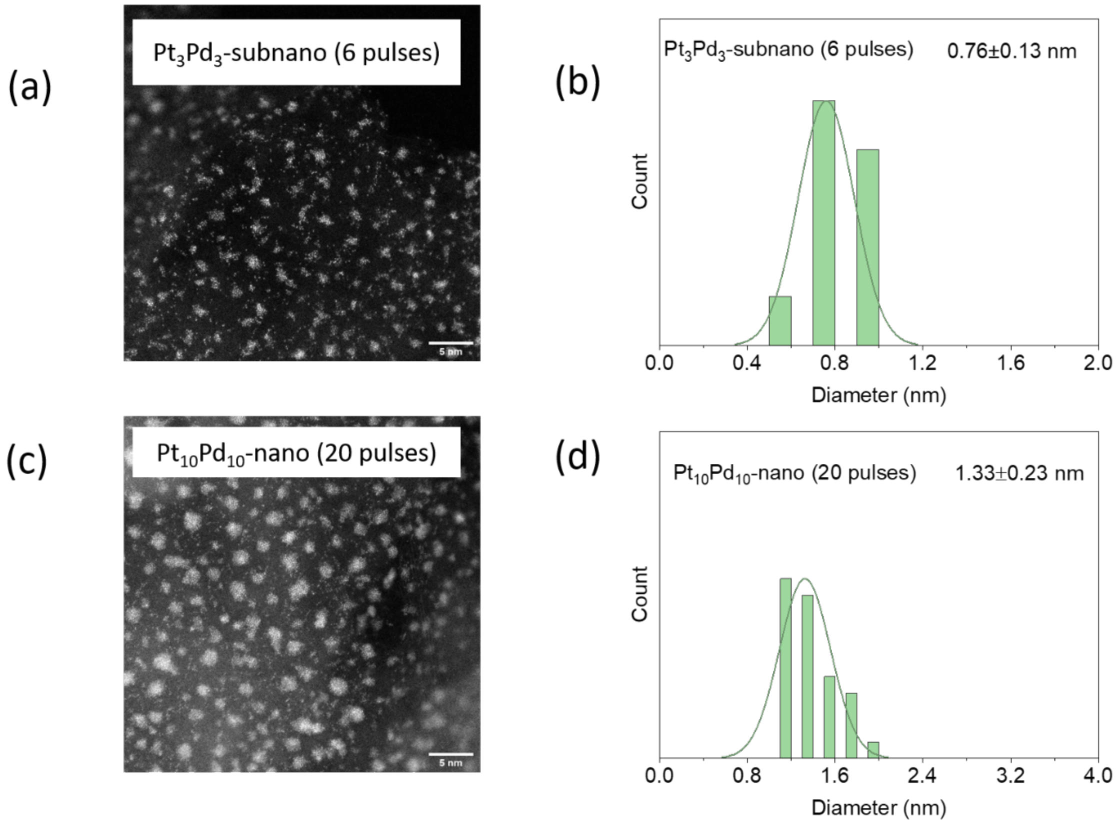

Figure S3. ADF-STEM and particle size histograms of Pt_3Pd_3 SNPs and $Pt_{10}Pd_{10}$ NPs.

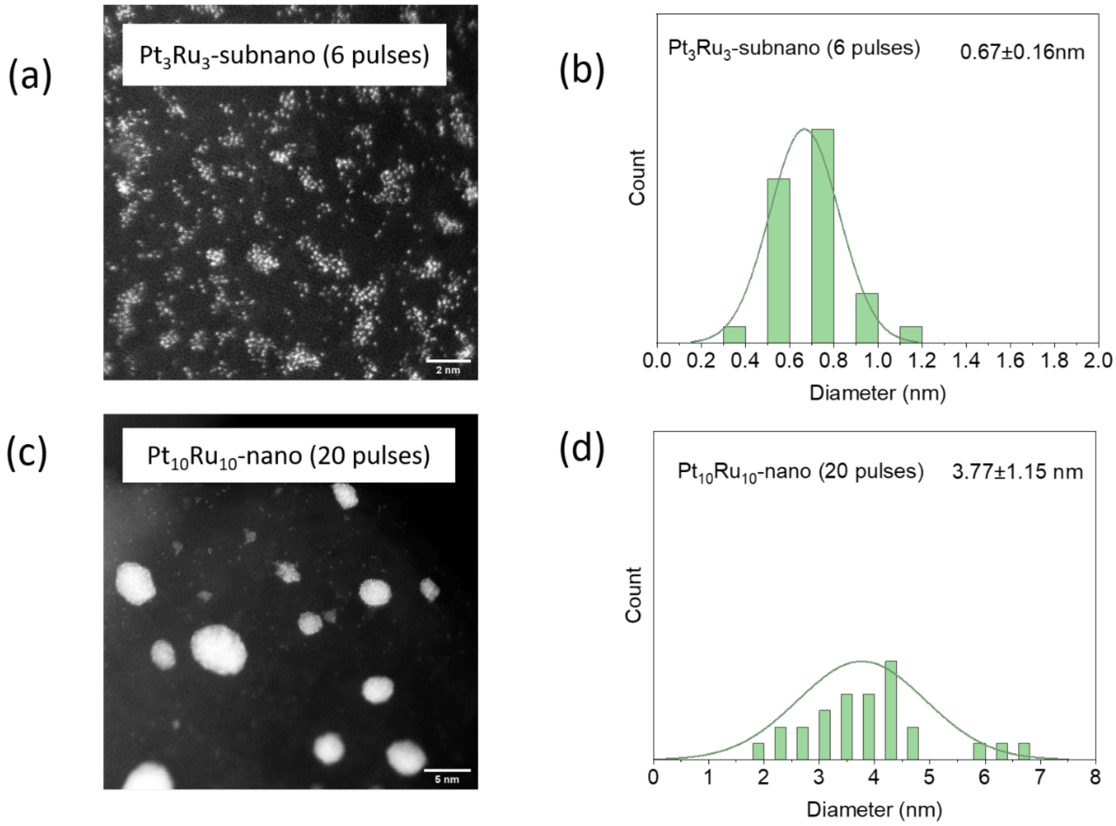

Figure S4. ADF-STEM and particle size histograms of Pt_3Ru_3 SNPs and $Pt_{10}Ru_{10}$ NPs.

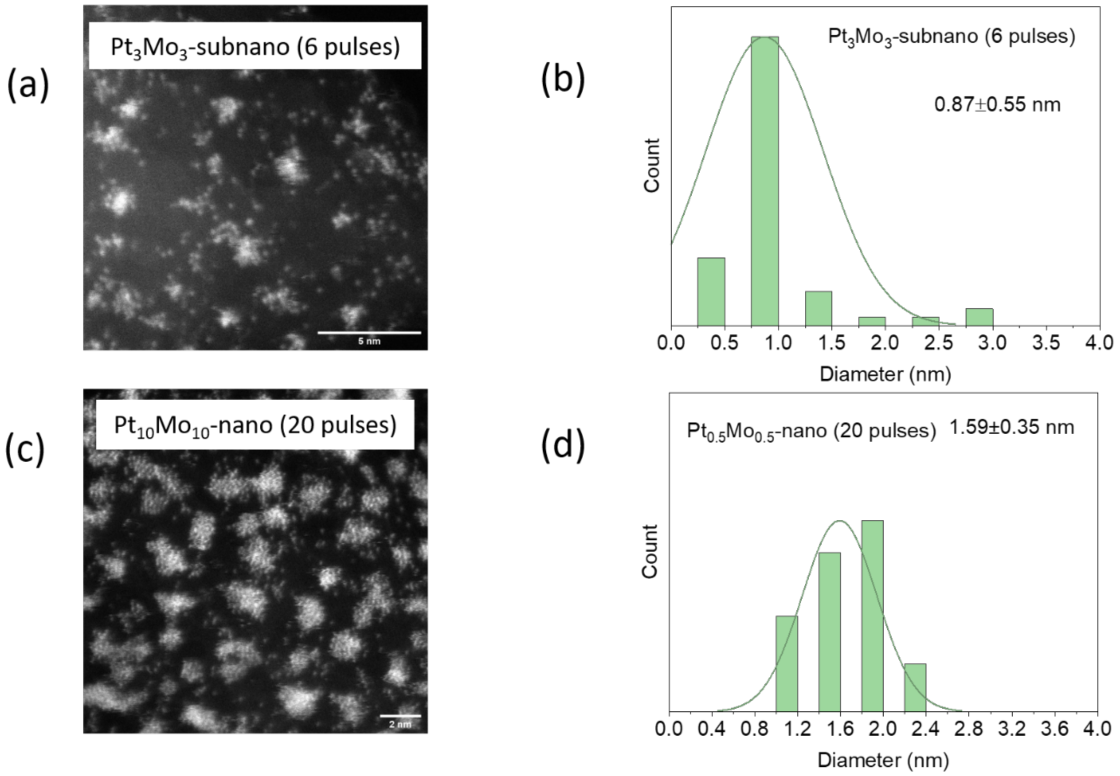

Figure S5. ADF-STEM and particle size histograms of Pt_3Mo_3 SNPs and $Pt_{10}Mo_{10}$ NPs.

.

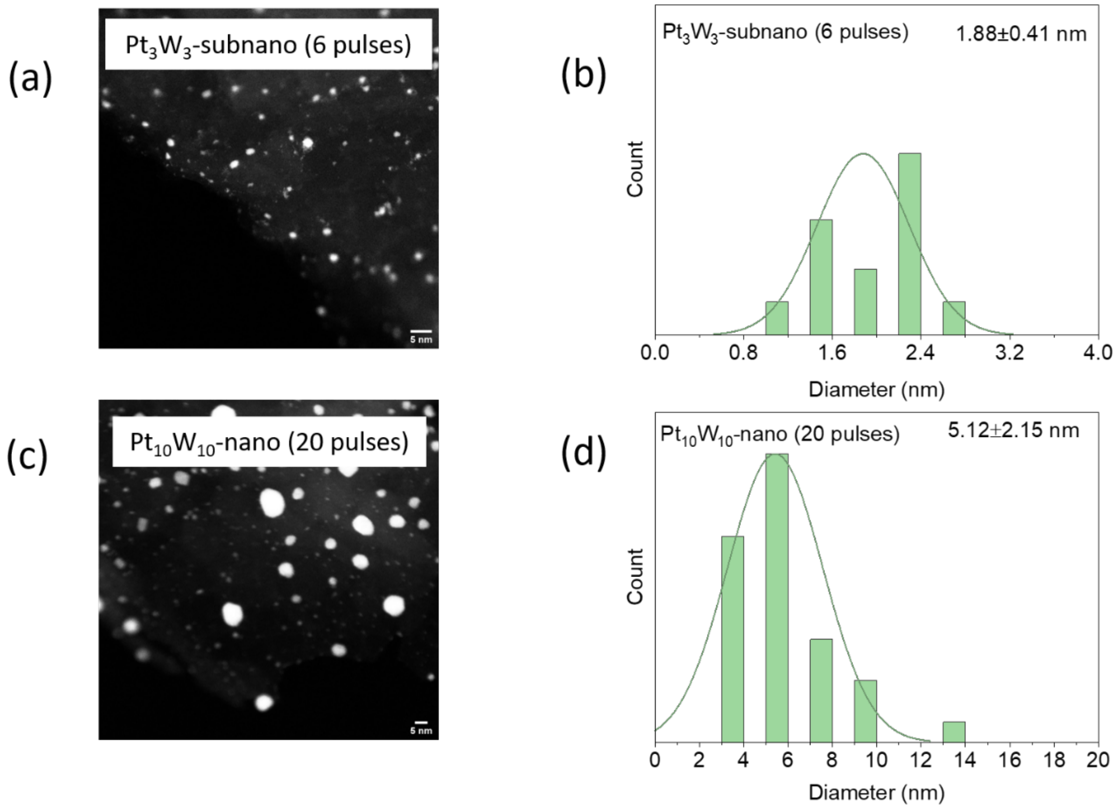

Figure S6. ADF-STEM and particle size histograms of Pt_3W_3 SNPs and $Pt_{10}W_{10}$ NPs.

.

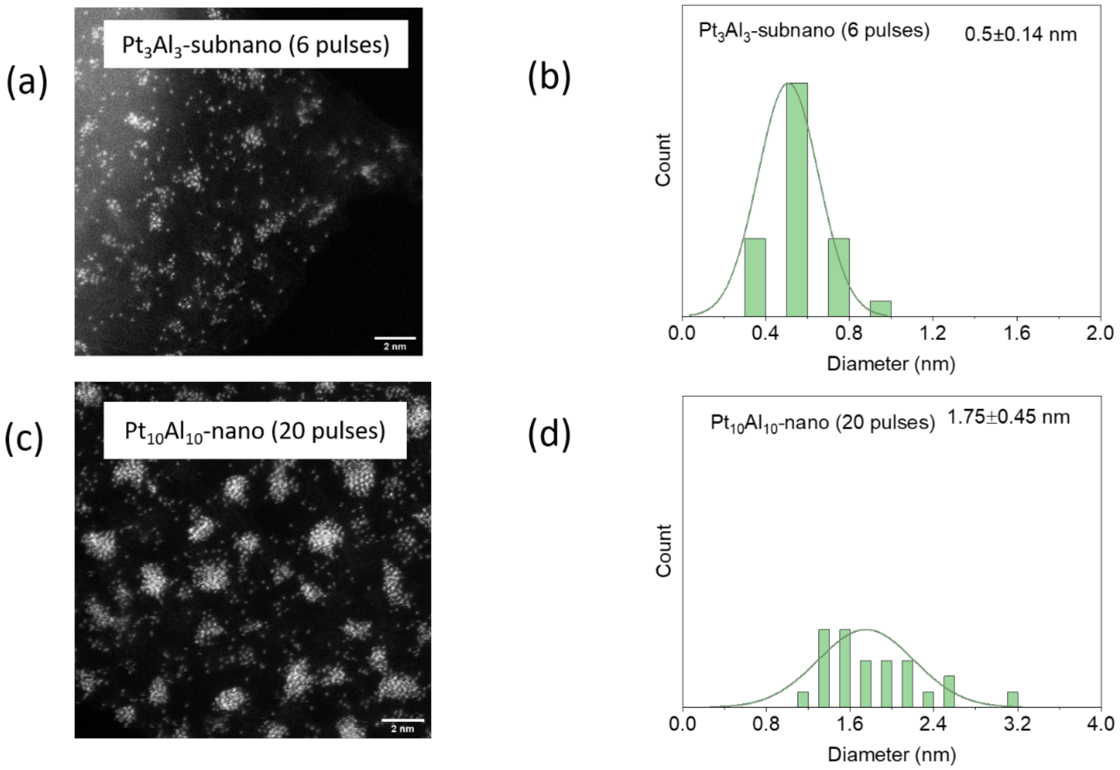

Figure S7. ADF-STEM and particle size histograms of Pt_3Al_3 SNPs and $Pt_{10}Al_{10}$ NPs.

.

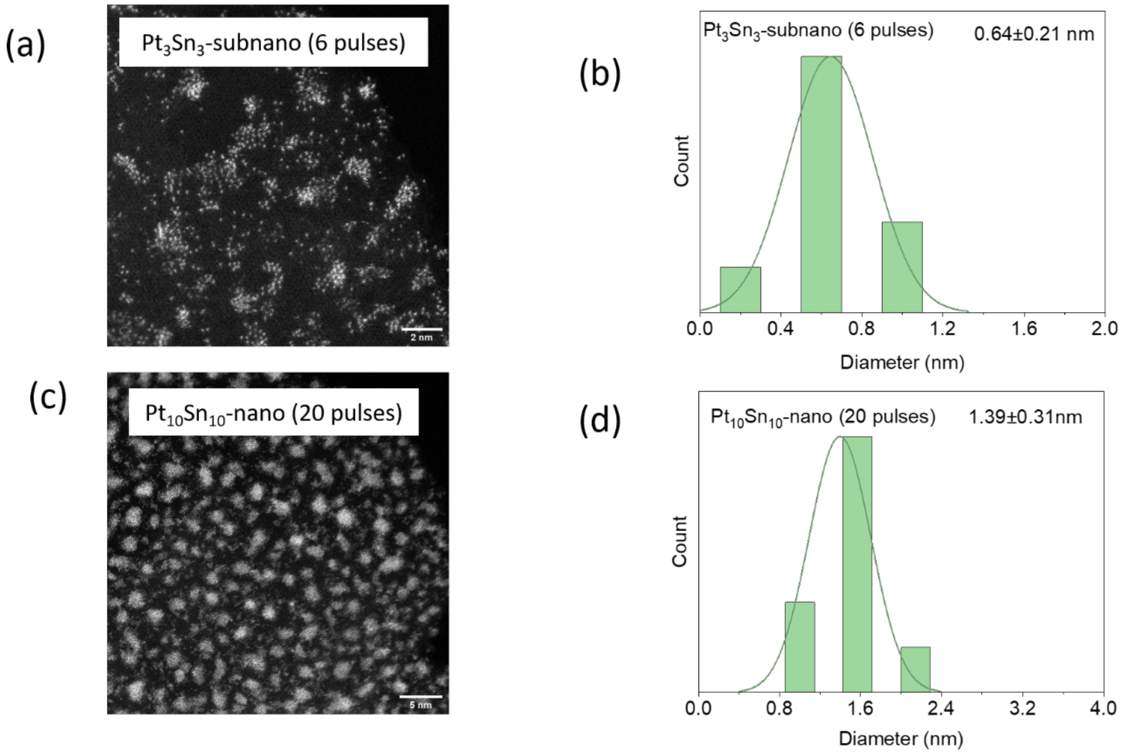

Figure S8. ADF-STEM and particle size of Pt_3Sn_3 SNPs and $Pt_{10}Sn_{10}$ NPs.

.

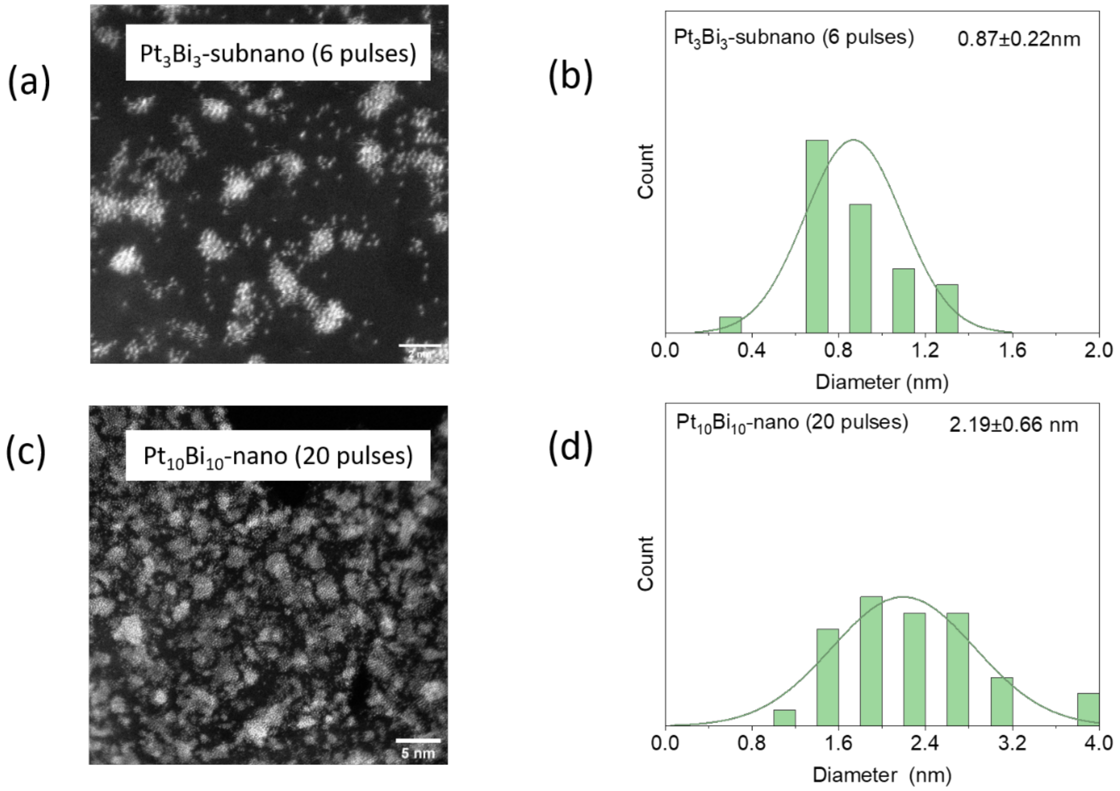

Figure S9. ADF-STEM and particle size histograms of Pt_3Bi_3 SNPs and $Pt_{10}Bi_{10}$ NPs

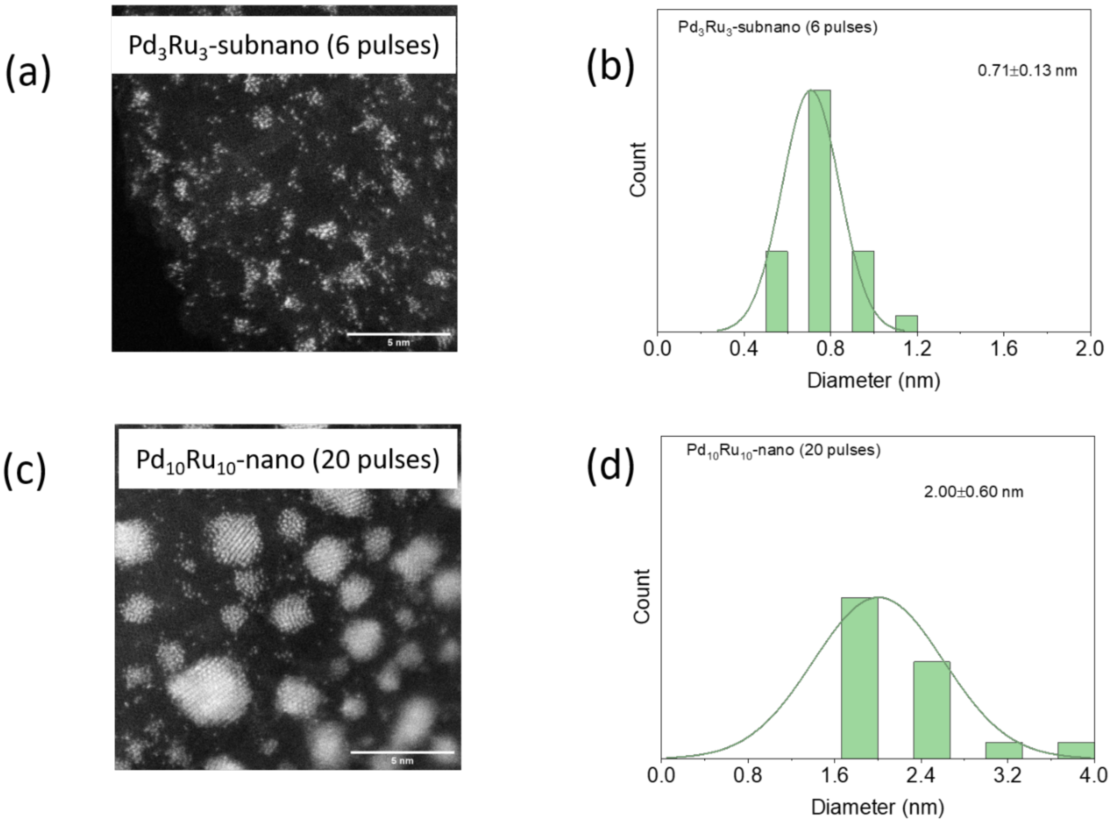

Figure S10. ADF-STEM and particle size histograms of Pd_3Ru_3 SNPs and $Pd_{10}Ru_{10}$ NPs

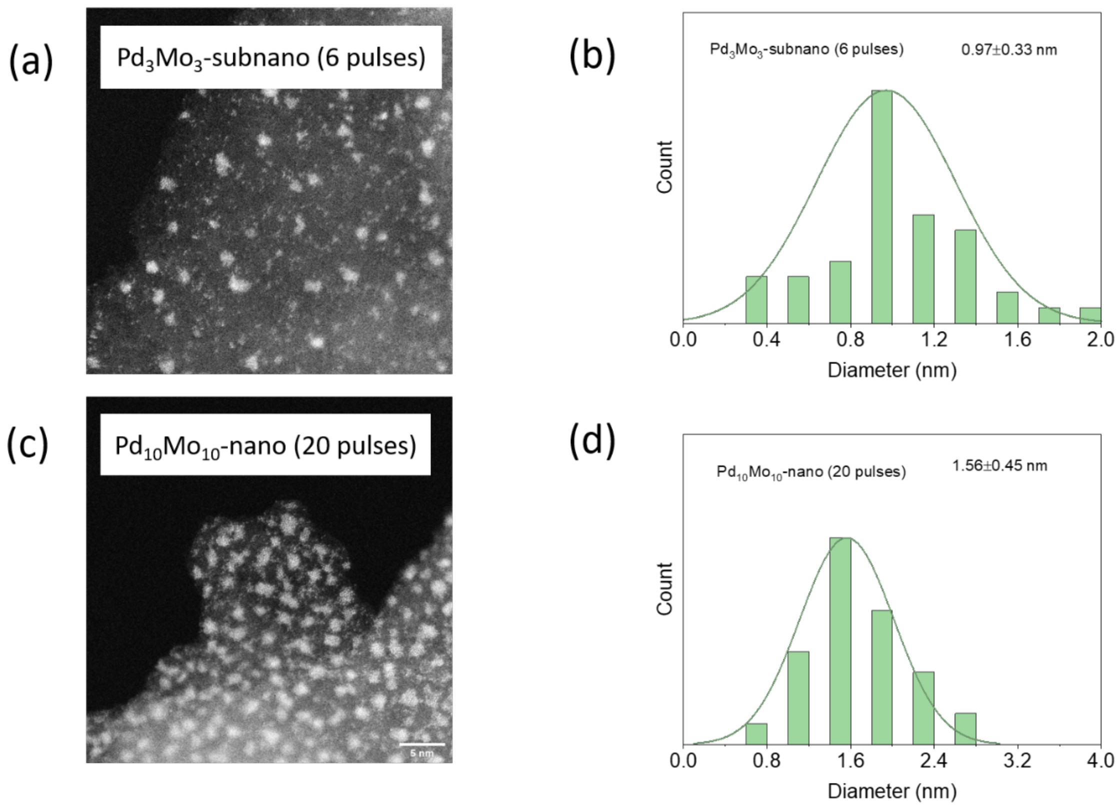

Figure S11. ADF-STEM and particle size histograms of Pd_3Mo_3 SNPs and $Pd_{10}Mo_{10}$ NPs

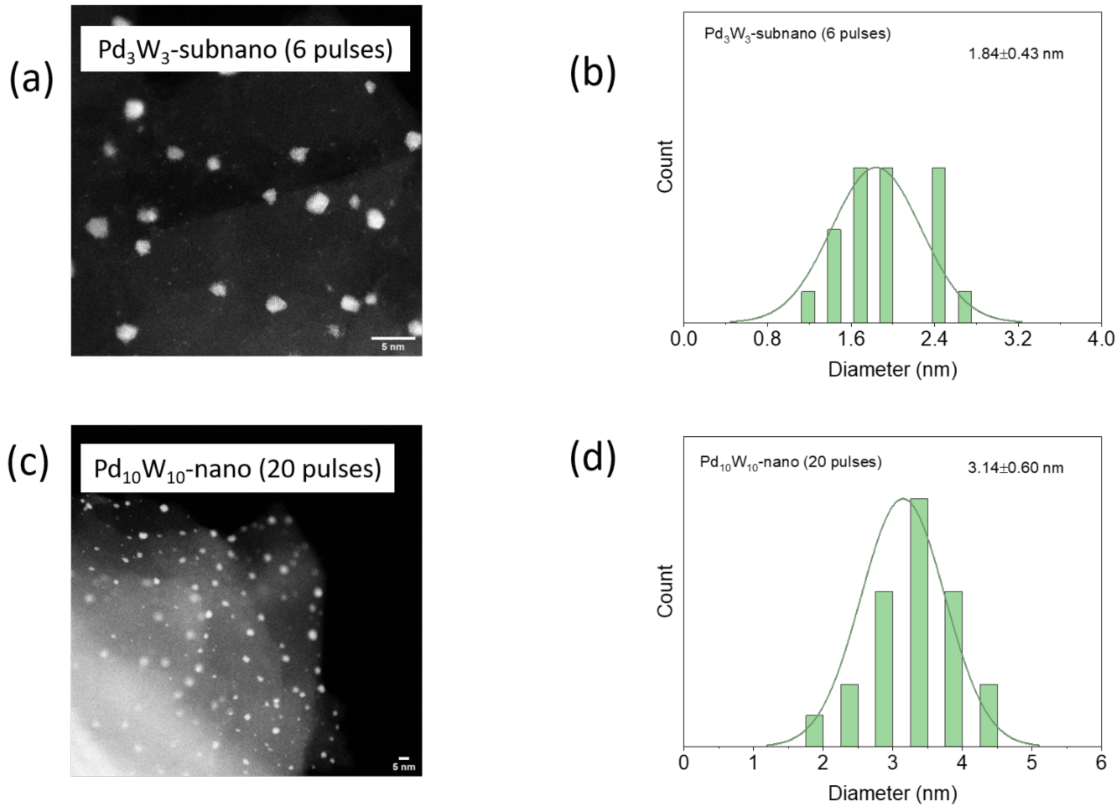

Figure S12. ADF-STEM and particle size histograms of Pd_3W_3 SNPs and $Pd_{10}W_{10}$ NPs.

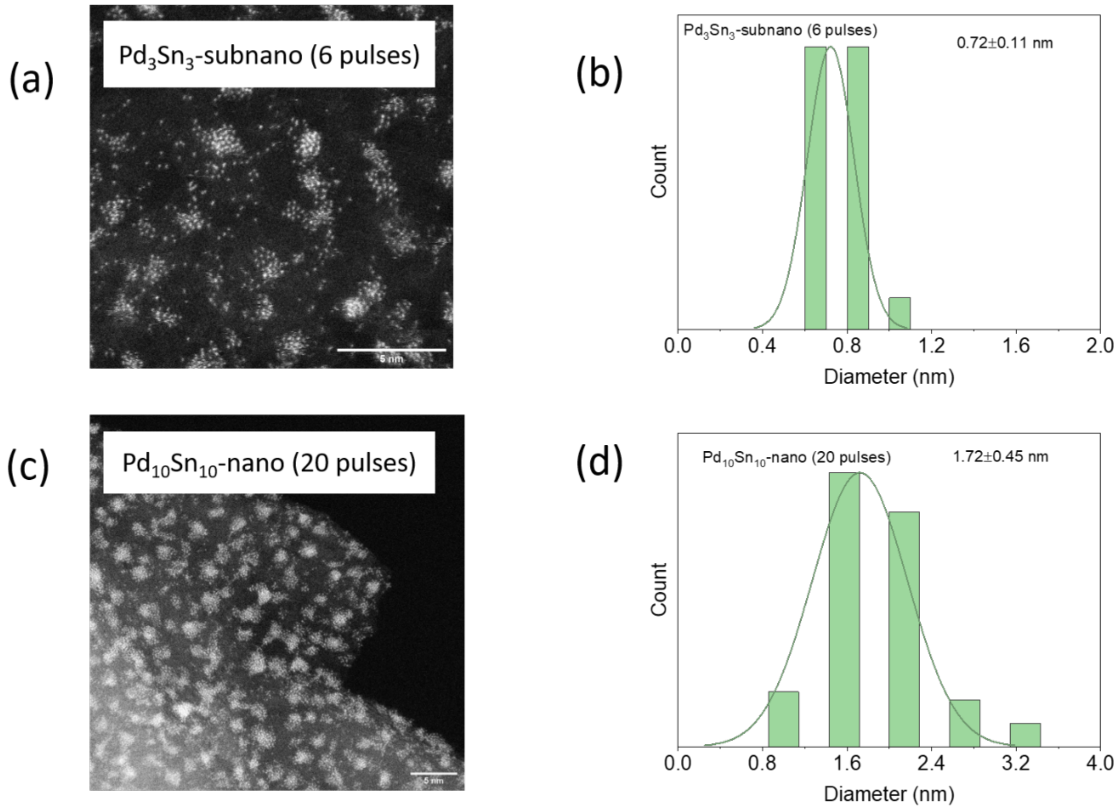

Figure S13. ADF-STEM and particle size histograms of Pd_3Sn_3 SNPs and $Pd_{10}Sn_{10}$ NPs.

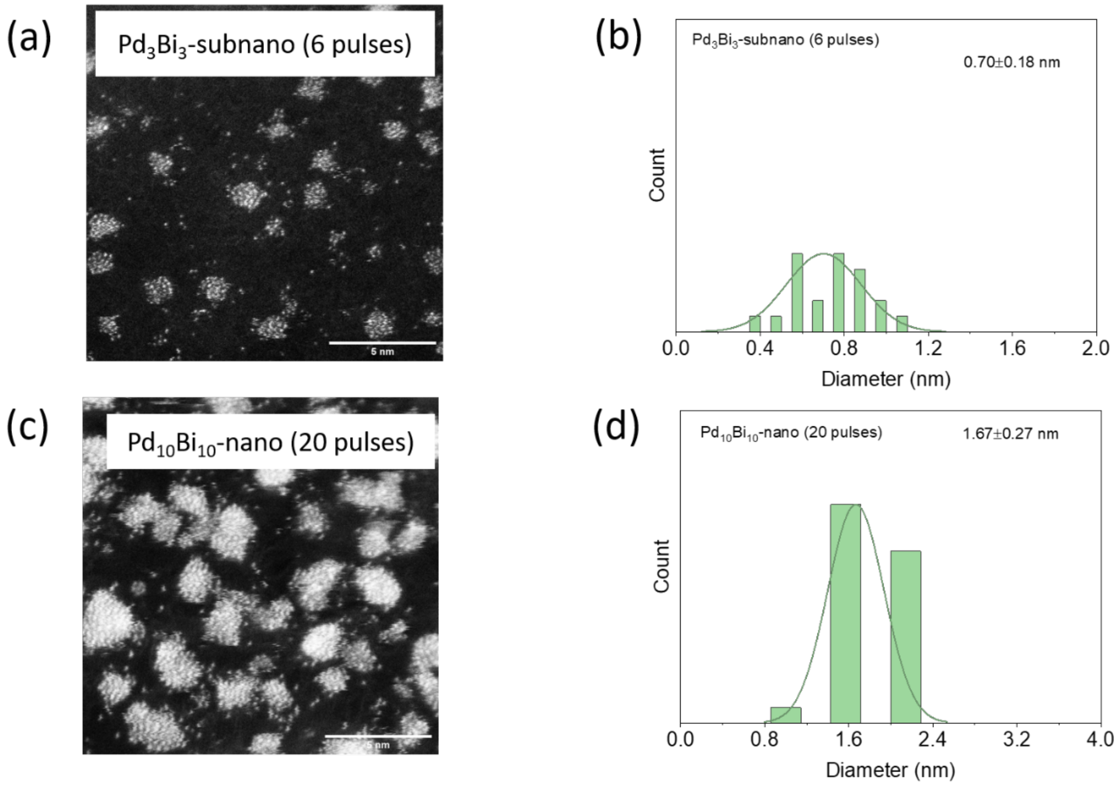

Figure S14. ADF-STEM and particle size histograms of Pd_3Bi_3 SNPs and $Pd_{10}Bi_{10}$ NPs.

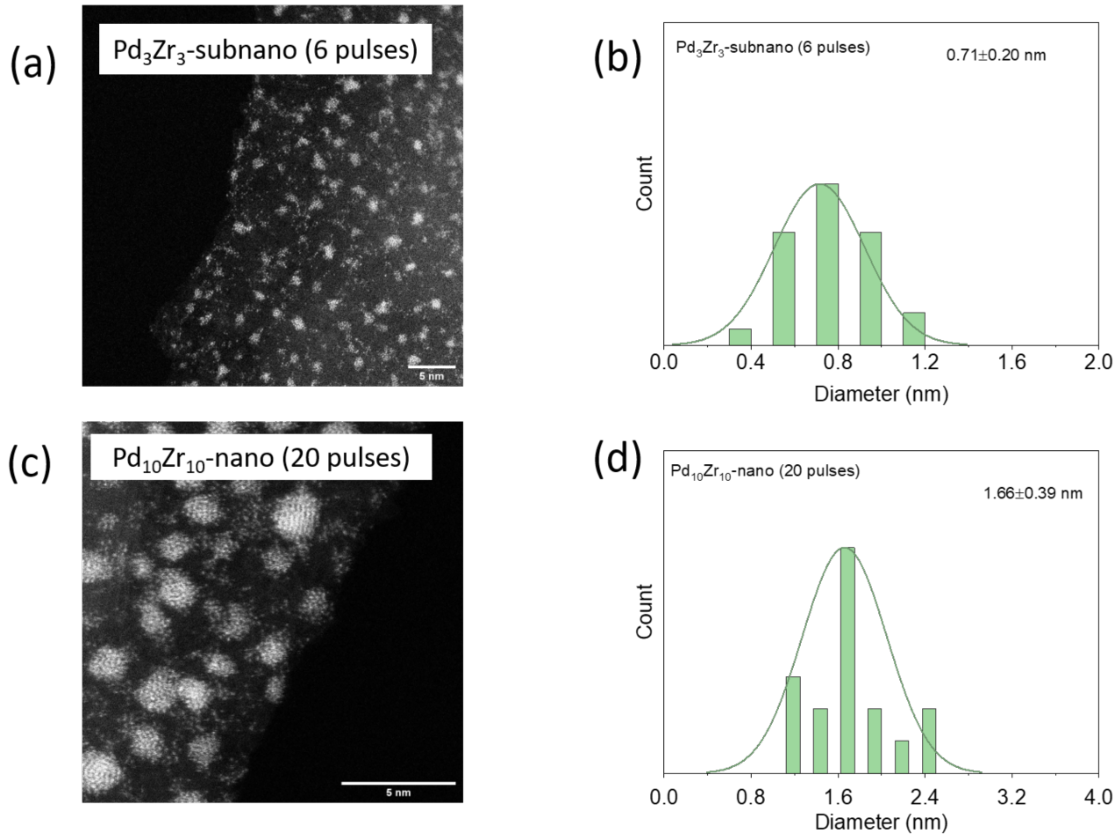

Figure S15. ADF-STEM and particle size histograms of Pd_3Zr_3 SNPs and $Pd_{10}Zr_{10}$ NPs.

.

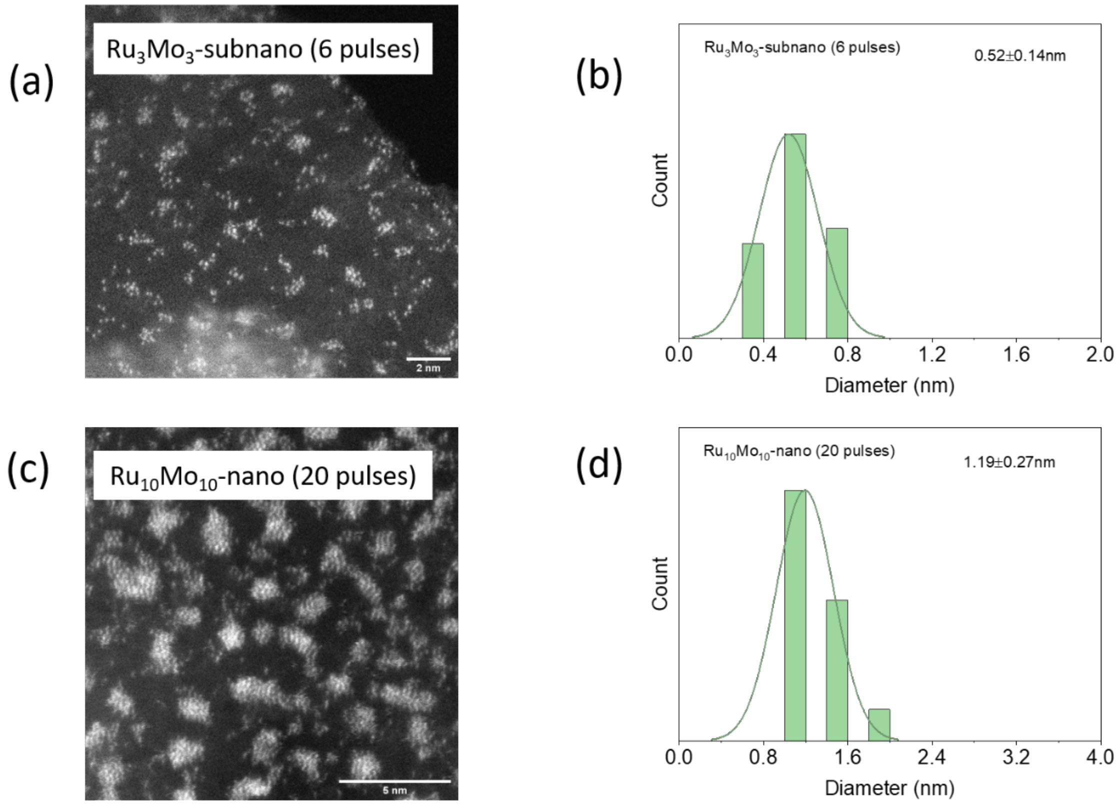

Figure S16. ADF-STEM and particle size histograms of Ru_3Mo_3 SNPs and $Ru_{10}Mo_{10}$ NPs.

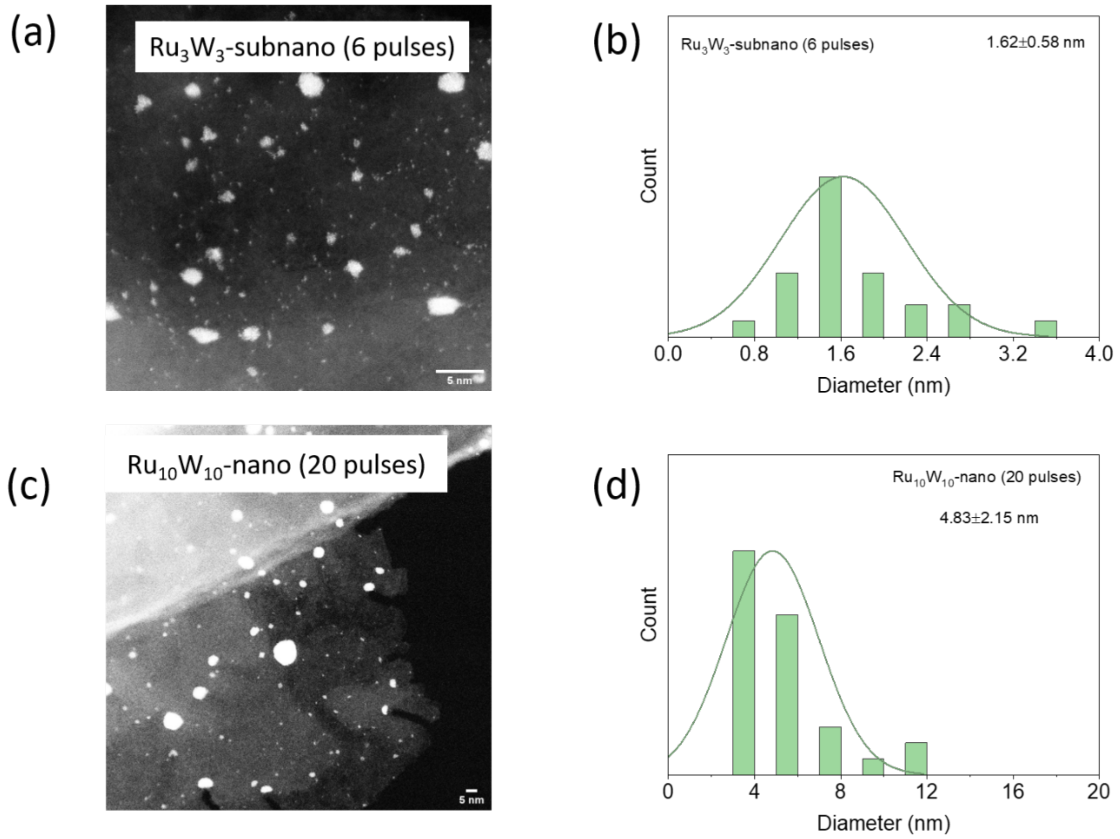

Figure S17. ADF-STEM and particle size histograms of Ru_3W_3 SNPs and $Ru_{10}W_{10}$ NPs.

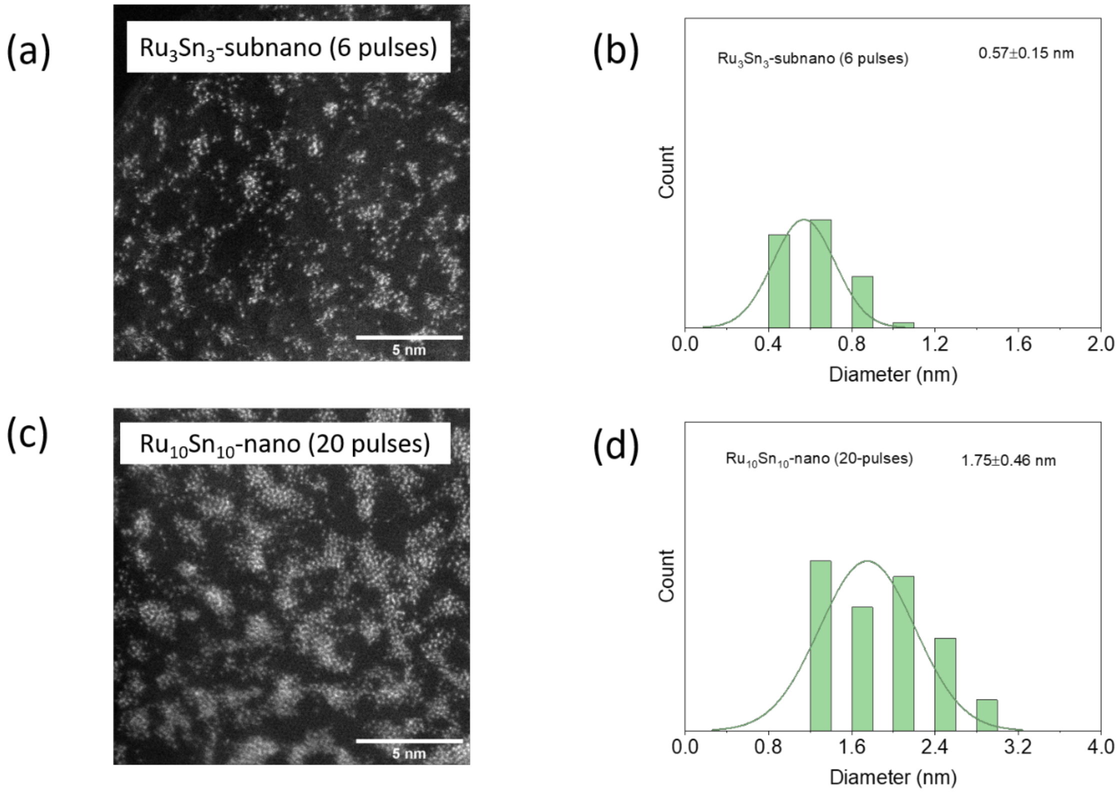

Figure S18. ADF-STEM and particle size histograms of Ru_3Sn_3 SNPs and $Ru_{10}Sn_{10}$ NPs.

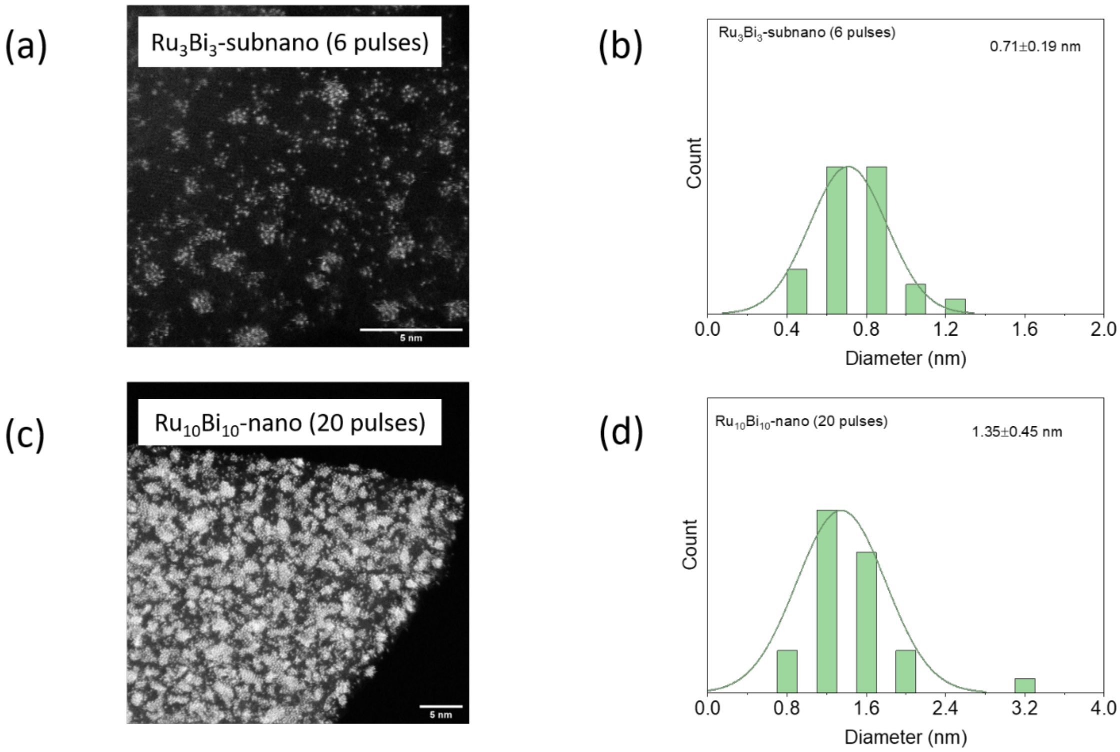

Figure S19. ADF-STEM and particle size histograms of Ru_3Bi_3 SNPs and $Ru_{10}Bi_{10}$ NPs.

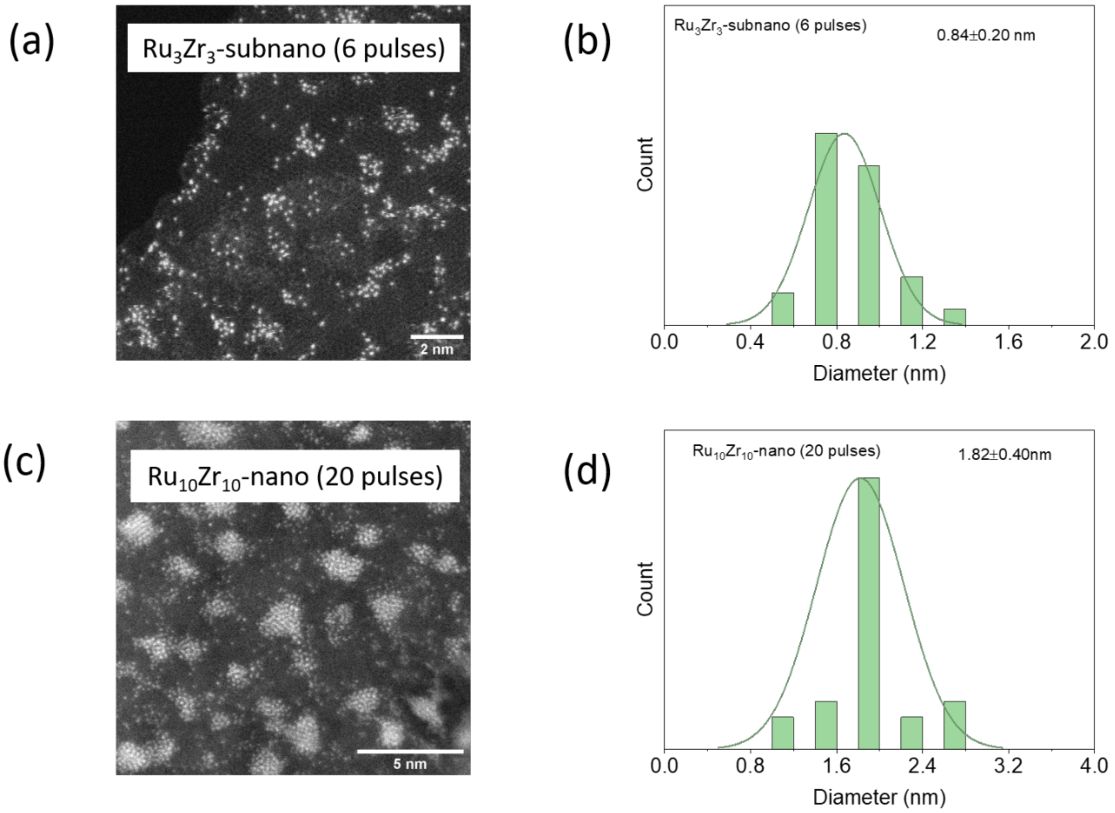

Figure S20. ADF-STEM and particle size histograms of Ru_3Zr_3 SNPs and $Ru_{10}Zr_{10}$ NPs.

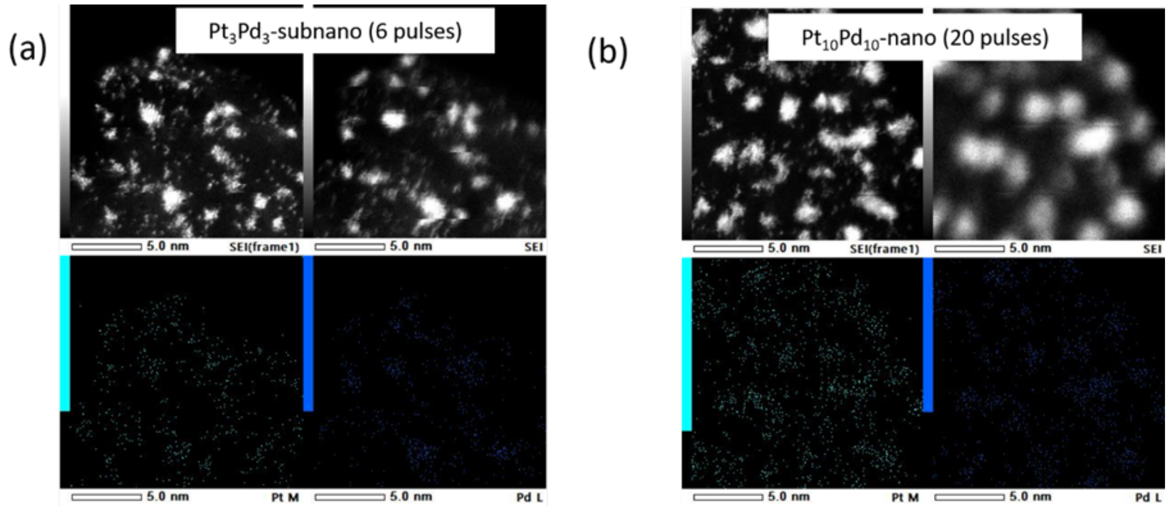

Figure S21. Elemental mapping of Pt_3Pd_3 SNPs and $Pt_{10}Pd_{10}$ NPs.

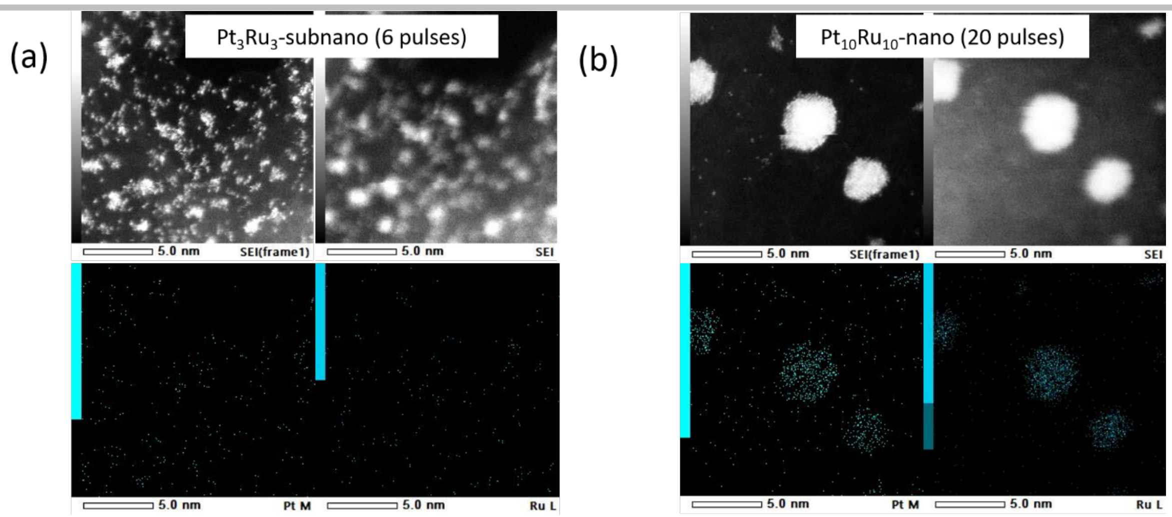

Figure S22. Elemental mapping of Pt_3Ru_3 SNPs and $Pt_{10}Ru_{10}$ NPs

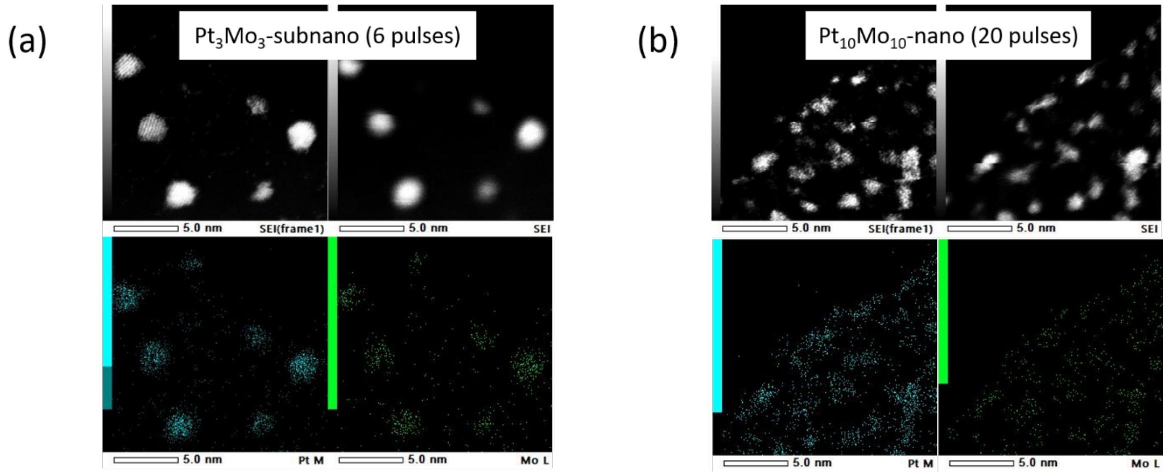

Figure S23. Elemental mapping of Pt_3Mo_3 SNPs and $Pt_{10}Mo_{10}$ NPs.

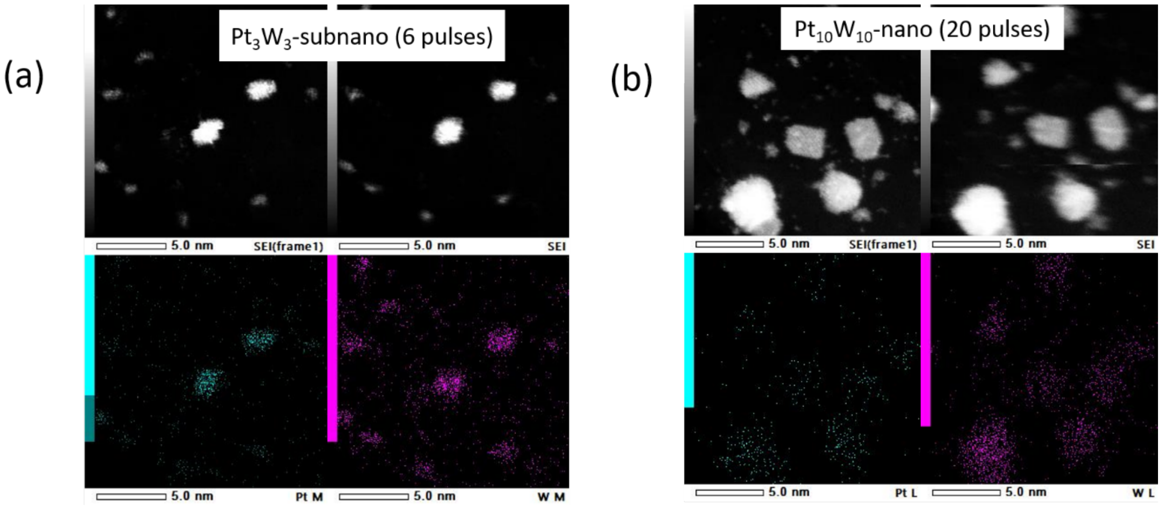

Figure S24. Elemental mapping of Pt_3W_3 SNPs and $Pt_{10}W_{10}$ NPs.

.

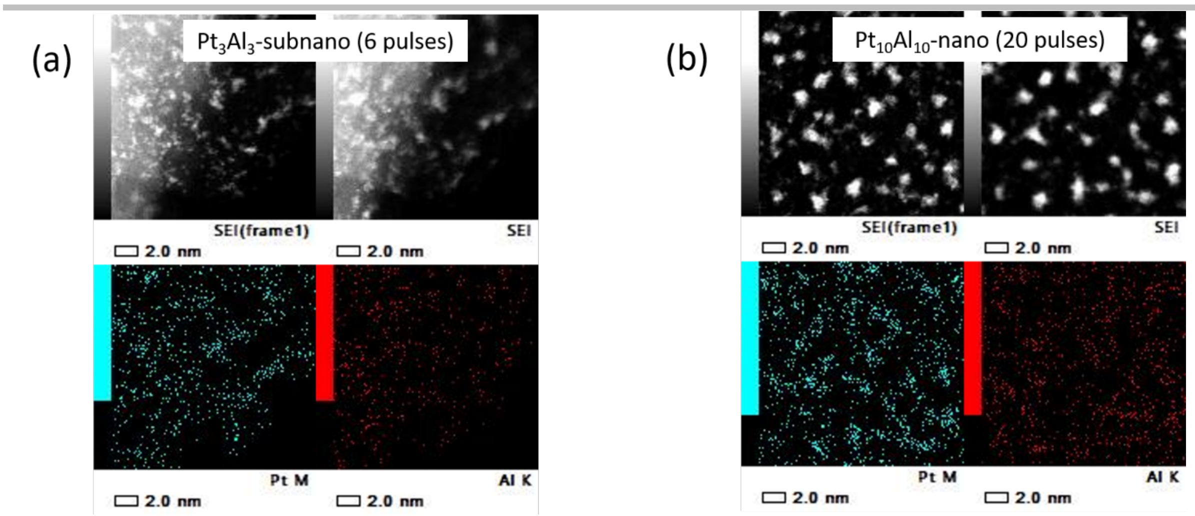

Figure S25. Elemental mapping of Pt_3Al_3 SNPs and $Pt_{10}Al_{10}$ NPs.

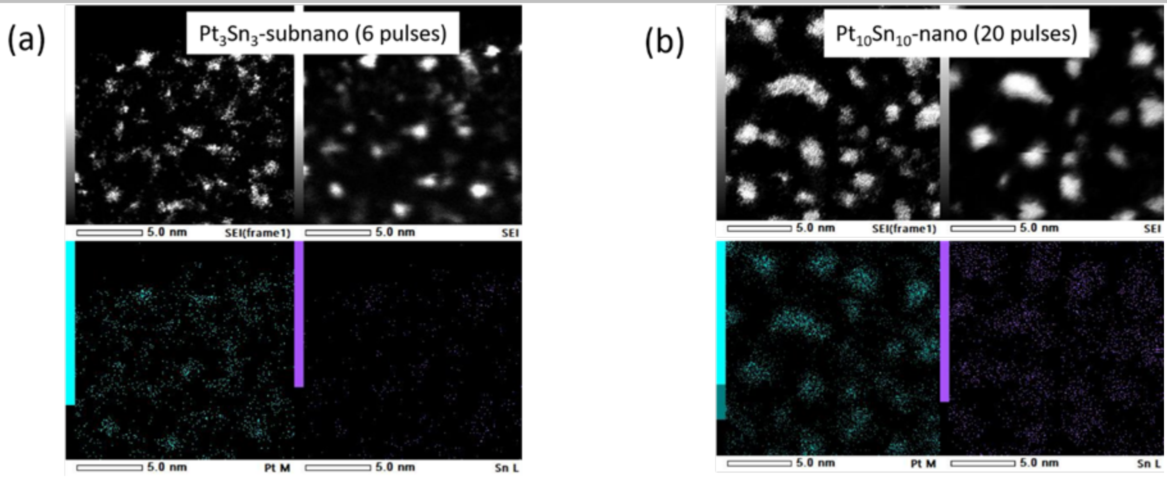

Figure S26. Elemental mapping of Pt_3Sn_3 SNPs and $Pt_{10}Sn_{10}$ NPs.

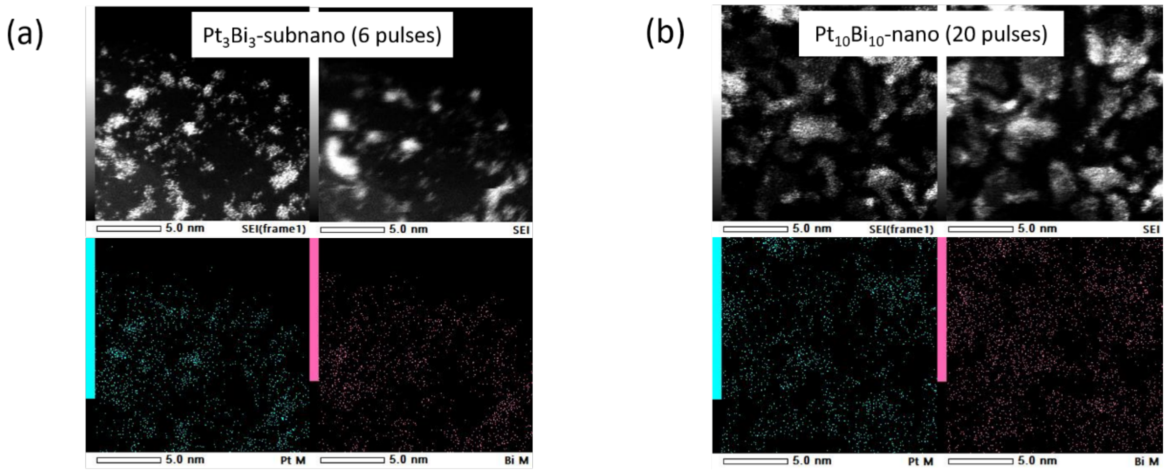

Figure S27. Elemental mapping of Pt_3Bi_3 SNPs and $Pt_{10}Bi_{10}$ NPs.

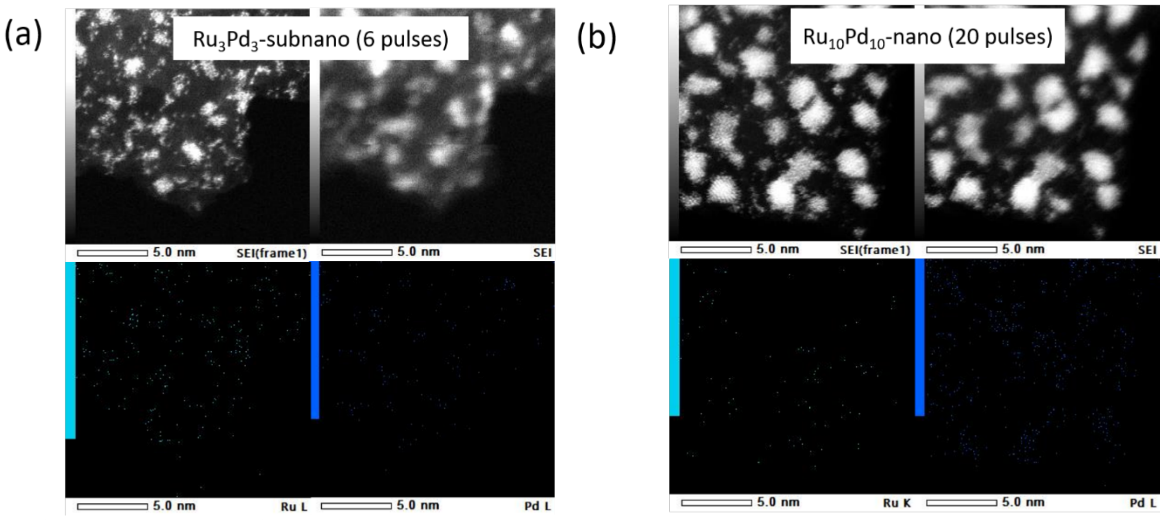

Figure S28. Elemental mapping of Pd_3Ru_3 SNPs and $Pd_{10}Ru_{10}$ NPs.

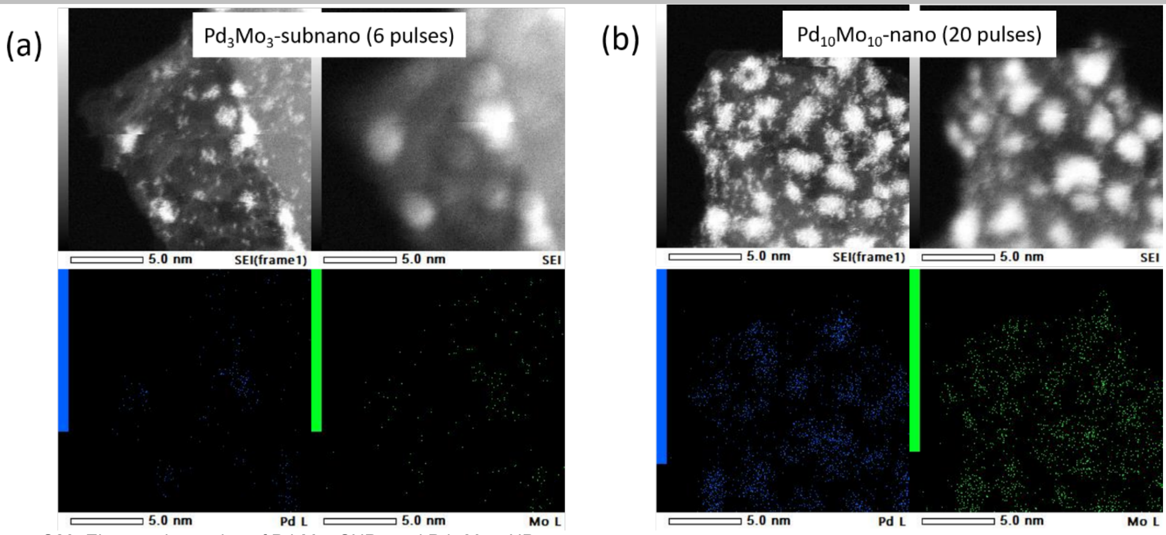

Figure S29. Elemental mapping of Pd_3Mo_3 SNPs and $Pd_{10}Mo_{10}$ NPs.

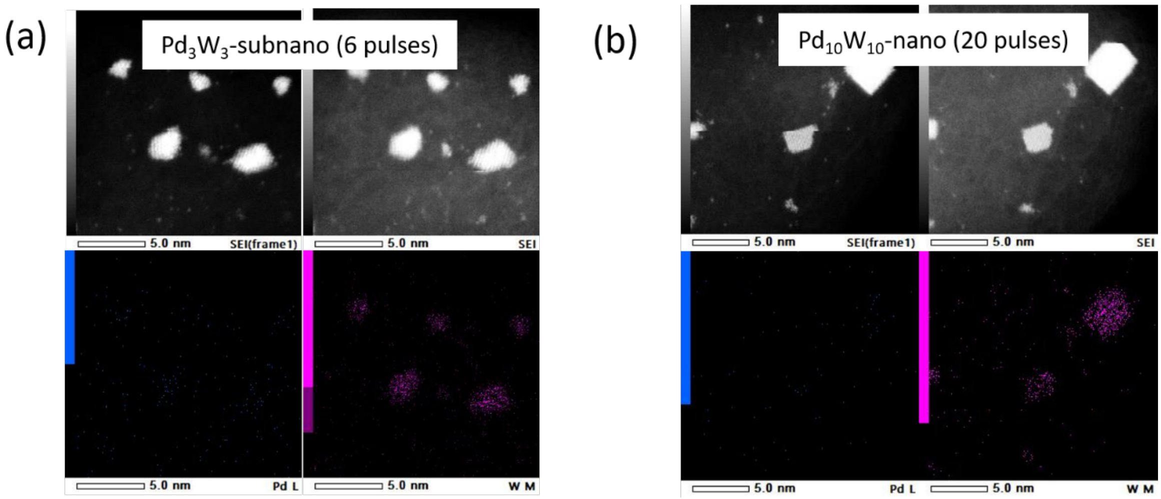

Figure S30. Elemental mapping of Pd_3W_3 SNPs and $Pd_{10}W_{10}$ NPs.

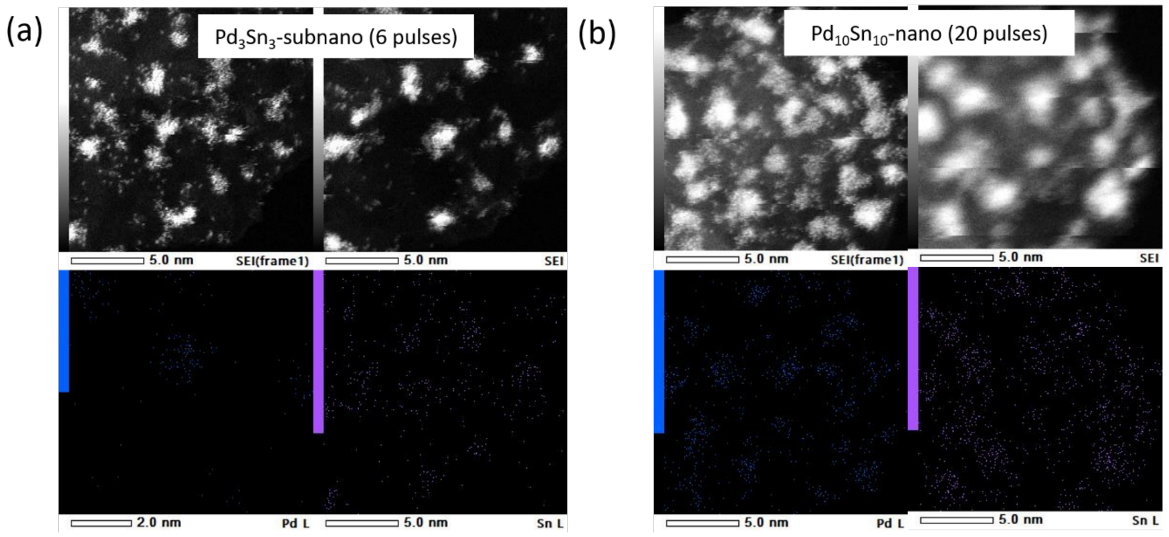

Figure S31. Elemental mapping of Pd_3Sn_3 SNPs and $Pd_{10}Sn_{10}$ NPs.

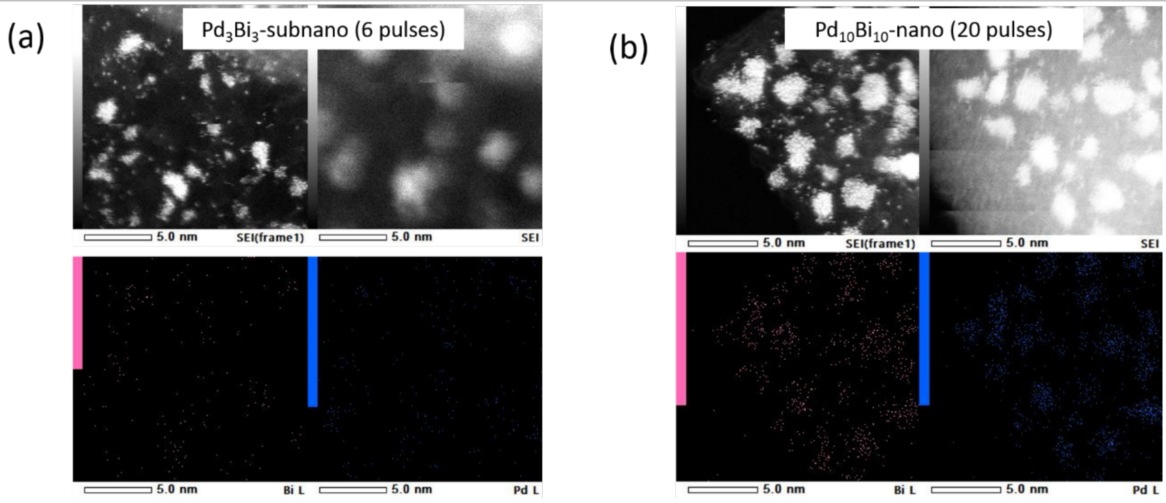

Figure S32. Elemental mapping of Pd_3Bi_3 SNPs and $Pd_{10}Bi_{10}$ NPs.

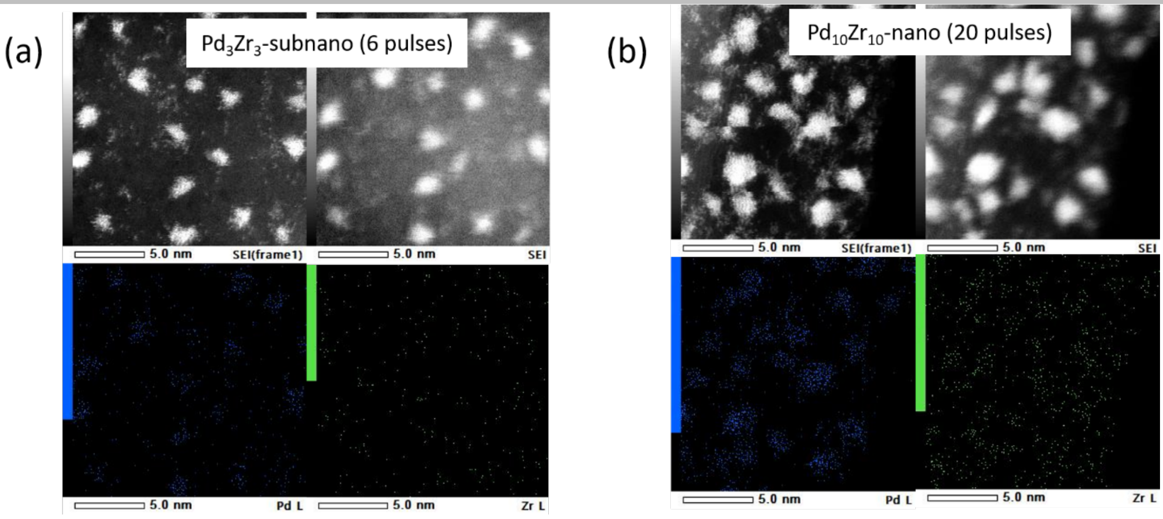

Figure S33. Elemental mapping of Pd_3Zr_3 SNPs and $Pd_{10}Zr_{10}$ NPs.

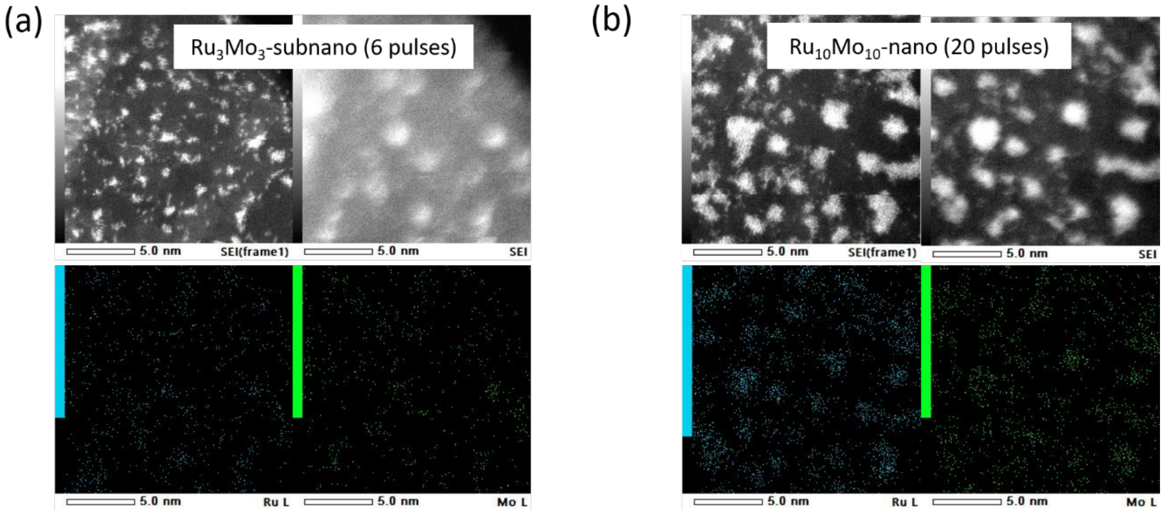

Figure S34. Elemental mapping of Ru_3Mo_3 SNPs and $Ru_{10}Mo_{10}$ NPs.

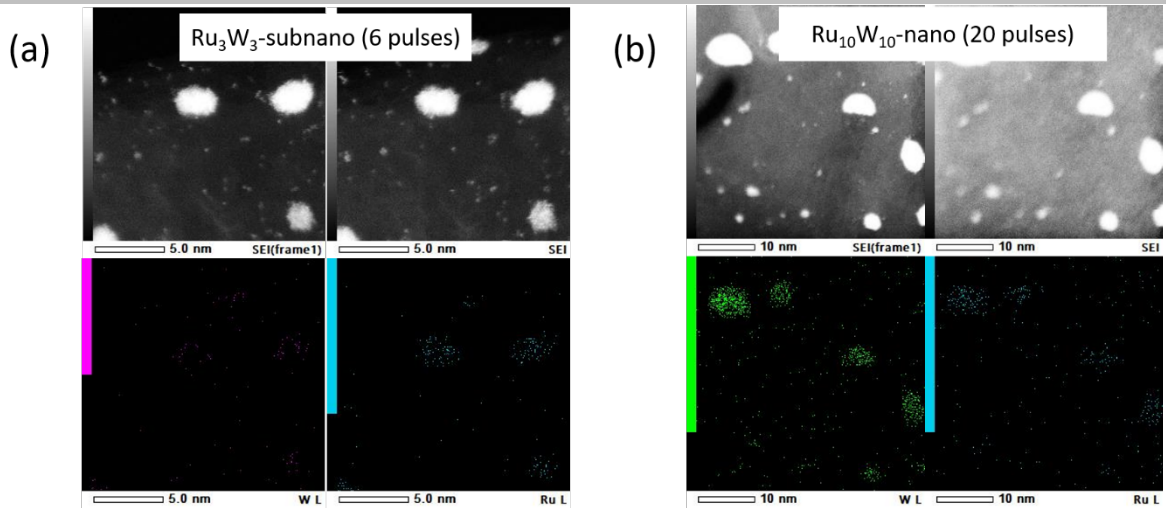

Figure S35. Elemental mapping of Ru_3W_3 SNPs and $Ru_{10}W_{10}$ NPs.

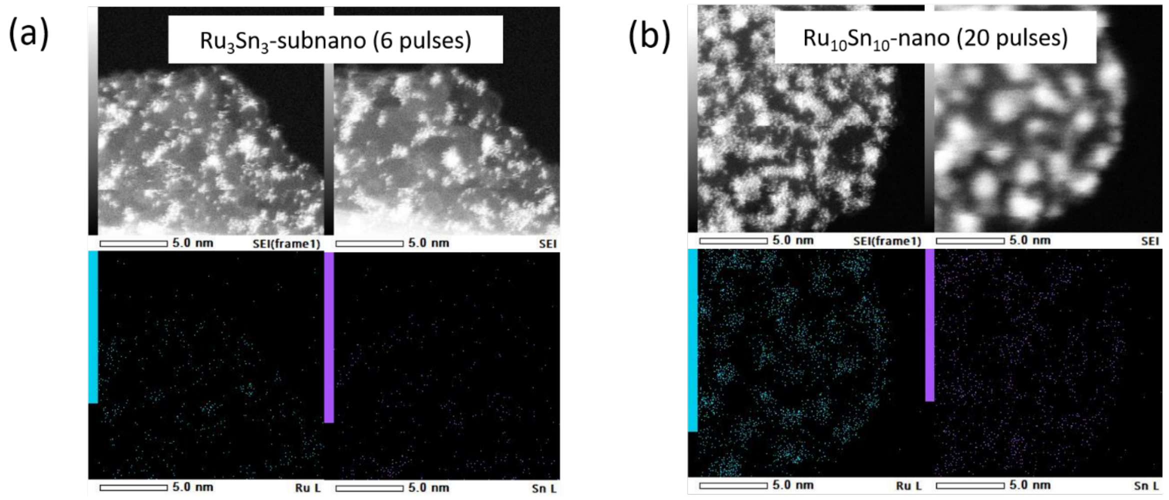

Figure S36. Elemental mapping of Ru_3Sn_3 SNPs and $Ru_{10}Sn_{10}$ NPs.

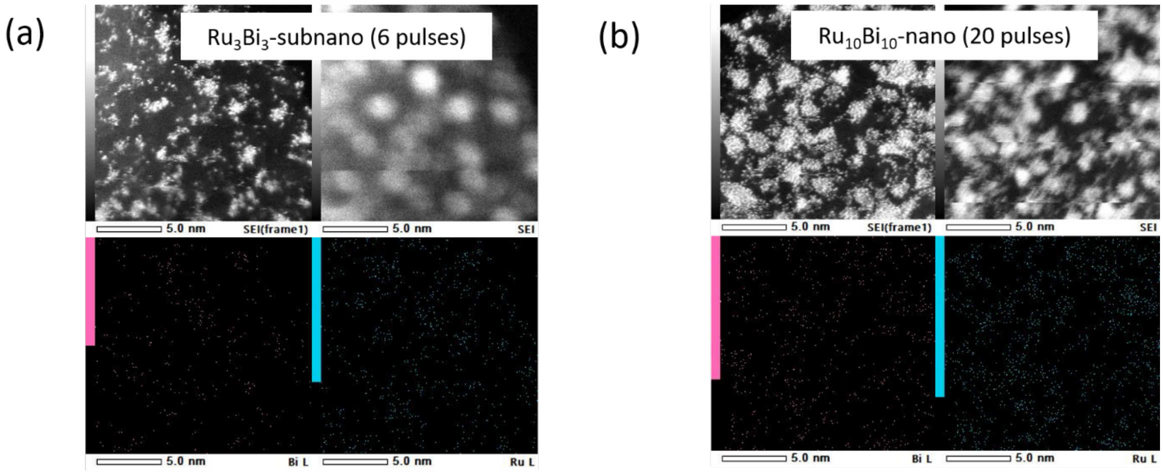

Figure S37. Elemental mapping of Ru_3Bi_3 SNPs and $Ru_{10}Bi_{10}$ NPs.

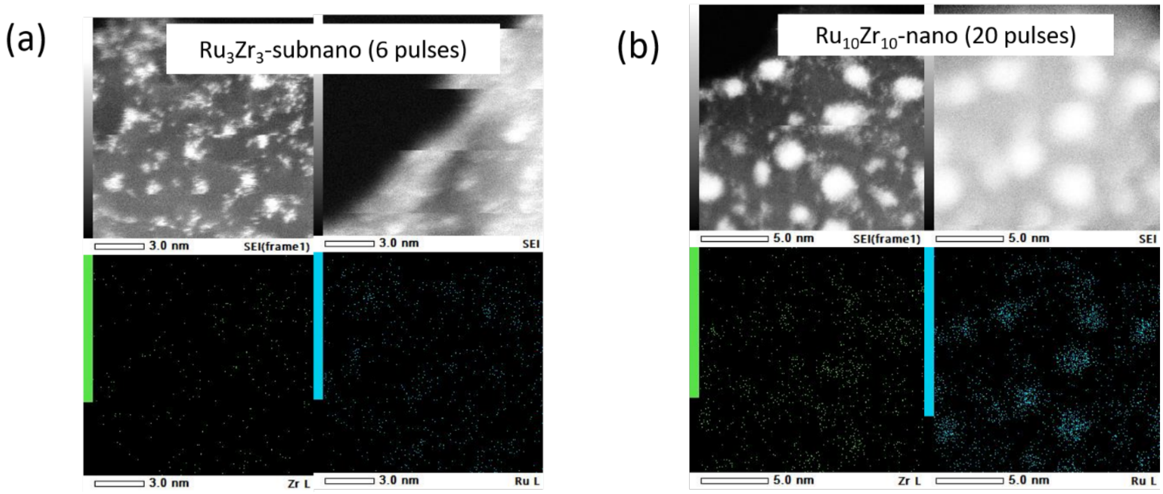

Figure S38. Elemental mapping of Ru_3Zr_3 SNPs and $Ru_{10}Zr_{10}$ NPs.

Section S4. X-ray photoelectron spectroscopy (XPS)

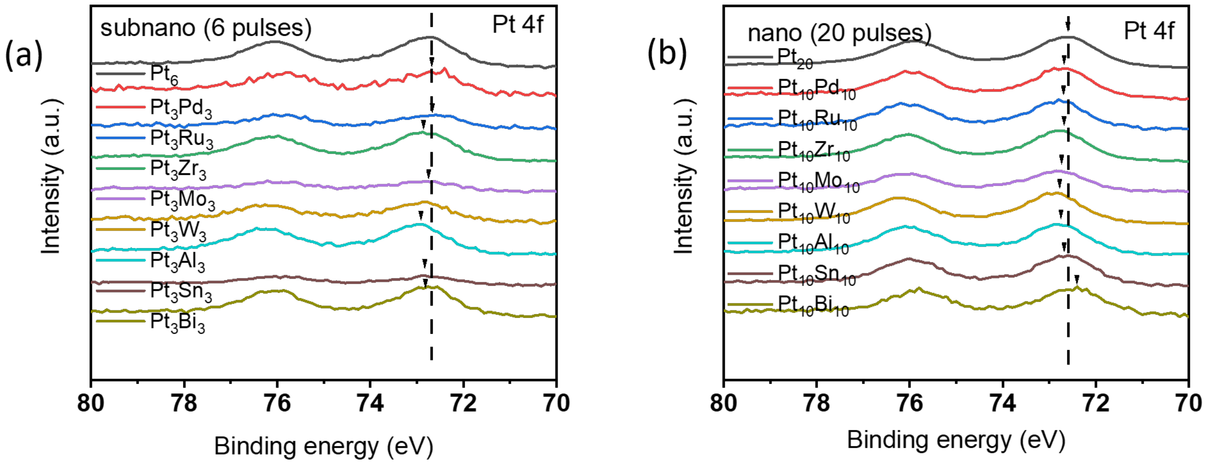

Figure S39. Pt 4*f* XPS of (a) Pt-based SNPs and (b) Pt-based NPs

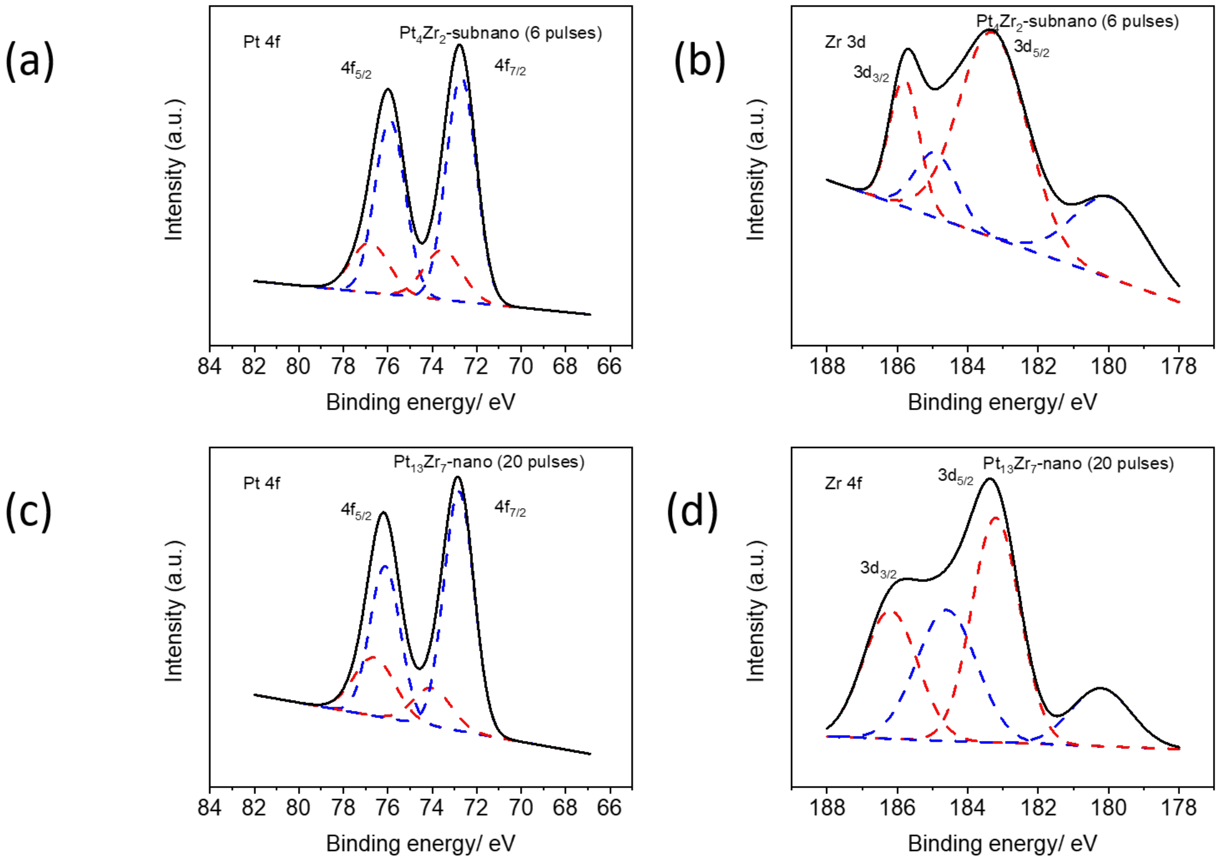

Figure S40. Pt 4f and Zr 3d XPS of (a, b) Pt_4Zr_2 SNPs and (c, d) $Pt_{13}Zr_7$ NPs.

Section S5. Electrochemical measurements

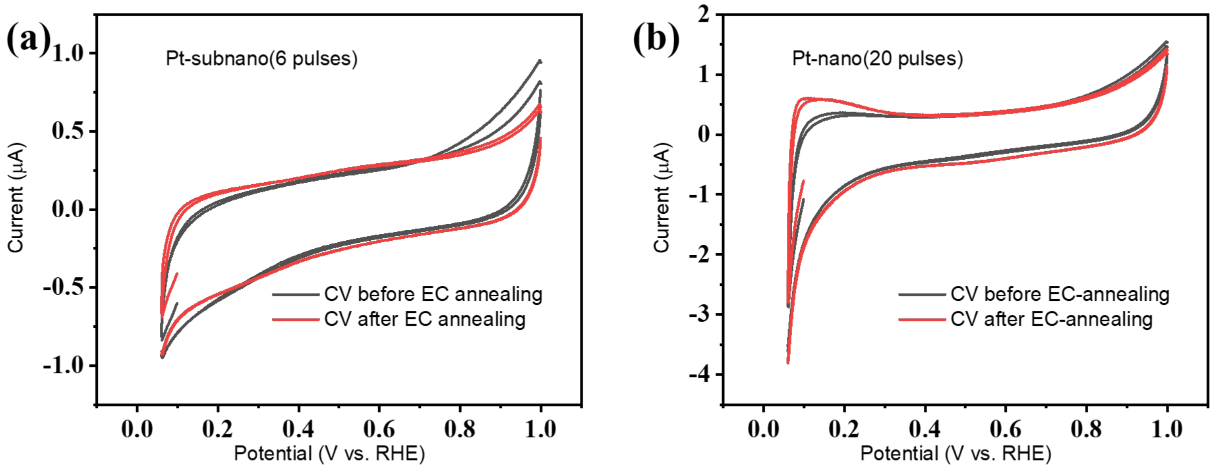

Figure S41. CV curves of Pt SNPs and NPs before and after EC annealing.

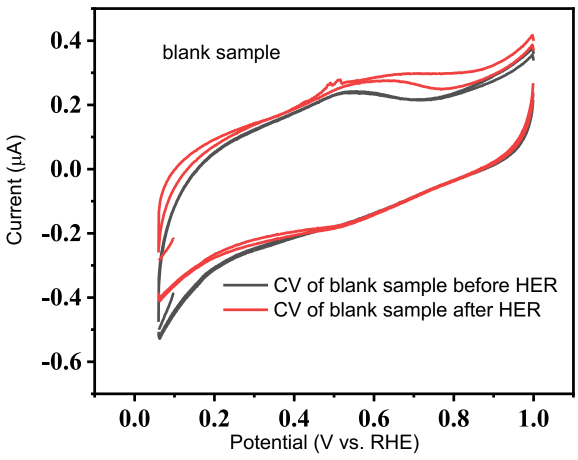

Figure S42. CV curves of blank sample before and after HER.

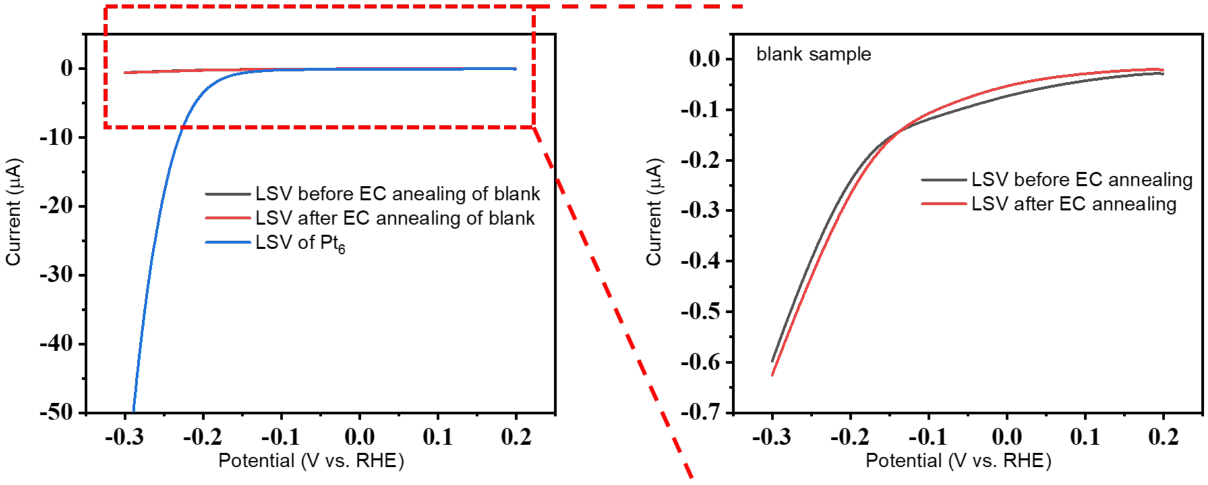

Figure S43. LSV curves of blank sample, blank sample after EC annealing and Pt_6 SNPs.

Table S2. Exchange current and Tafel slope for unary and bimetallic nanoparticles.

No.	M1	M2	M1 pulse numbers	M2 pulse numbers	Exchange current (μA)	Tafel slope ($mV\ dec^{-1}$)
1	Pt	0	20	0	22	37
2	Pd	0	20	0	3.78	53
3	Ru	0	20	0	1.703	68
4	Mo	0	20	0	0.1685	180
5	W	0	20	0	0.08112	129
6	Zr	0	20	0	0.06955	267
7	Al	0	20	0	1.687	47
8	Bi	0	20	0	0.1026	400
9	Sn	0	20	0	0.06021	305
10	Pt	Mo	10	10	0.2497	148
11	Pt	Zr	10	10	17.98	37.5
12	Pt	Pd	10	10	4.088	49
13	Pt	Al	10	10	0.146	90
14	Pt	Bi	10	10	12.6	40
15	Pt	Sn	10	10	1.158	80
16	Pt	Ru	10	10	14.31	44
17	Pt	W	10	10	0.5921	80
18	Pd	Mo	10	10	0.2988	83
19	Pd	Zr	10	10	1.883	65
20	Pd	W	10	10	0.1466	84
21	Pd	Al	10	10	0.2588	73
22	Pd	Bi	10	10	0.9183	84
23	Pd	Sn	10	10	0.2413	133
24	Pd	Ru	10	10	3.125	68
25	Ru	Mo	10	10	0.1889	64
26	Ru	Zr	10	10	0.255	64
27	Ru	W	10	10	0.1164	90
28	Ru	Al	10	10	0.1484	68
29	Ru	Bi	10	10	0.2373	68
30	Ru	Sn	10	10	0.1542	57
31	Mo	Sn	10	10	0.08118	237
32	Mo	Al	10	10	0.126	100
33	Mo	Bi	10	10	0.1209	80
34	Zr	Al	10	10	0.07754	300
35	Zr	Mo	10	10	0.1032	163
36	Zr	Sn	10	10	0.02288	160
37	Zr	Bi	10	10	0.1102	278
38	W	Al	10	10	0.07136	400
39	W	Bi	10	10	0.1138	400
40	W	Sn	10	10	0.07136	229
41	W	Mo	10	10	0.109	214

42	W	Zr	10	10	0.08102	170
43	Bi	Al	10	10	0.09727	150
44	Sn	Al	10	10	0.08393	200
45	Bi	Sn	10	10	0.07546	188

Table S3. Exchange current and Tafel slope for unary and bimetallic subnanoparticles.

No.	M1	M2	M1 pulse numbers	M2 pulse numbers	Exchange current (μA)	Tafel slope (mV dec^{-1})
1	Pt	0	6	0	0.1434	70
2	Pd	0	6	0	0.114	103
3	Ru	0	6	0	0.115	126
4	Mo	0	6	0	0.0492	137
5	W	0	6	0	0.07487	94
6	Zr	0	6	0	0.02964	167
7	Al	0	6	0	0.05322	140
8	Bi	0	6	0	0.0969	120
9	Sn	0	6	0	0.04713	318
10	Pt	Mo	3	3	0.07171	116
11	Pt	Zr	3	3	0.2357	74.3
12	Pt	W	3	3	0.05731	135
13	Pt	Pd	3	3	0.5054	62
14	Pt	Al	3	3	0.04891	125
15	Pt	Bi	3	3	0.1387	112
16	Pt	Sn	3	3	0.08957	389
17	Pt	Ru	3	3	0.4011	65
18	Pd	Mo	3	3	0.09875	109
19	Pd	Zr	3	3	0.1916	100
20	Pd	W	3	3	0.09359	86
21	Pd	Al	3	3	0.09297	97
22	Pd	Bi	3	3	0.1149	97
23	Pd	Sn	3	3	0.1094	153
24	Pd	Ru	3	3	0.1463	67
25	Ru	Mo	3	3	0.1335	107
26	Ru	Zr	3	3	0.1516	92
27	Ru	W	3	3	0.1092	109
28	Ru	Al	3	3	0.1245	100
29	Ru	Bi	3	3	0.115	110
30	Ru	Sn	3	3	0.1257	60
31	Mo	Sn	3	3	0.05465	33
32	Mo	Al	3	3	0.06632	100
33	Mo	Bi	3	3	0.1183	36
34	Mo	Zr	3	3	0.1133	200
35	Mo	W	3	3	0.1343	417
36	Zr	Bi	3	3	0.08145	171
37	Zr	Sn	3	3	0.04352	114.3
38	Zr	W	3	3	0.08446	147
39	Zr	Al	3	3	0.07537	117
40	Sn	Bi	3	3	0.07771	187
41	Sn	Al	3	3	0.09526	182

42	Sn	W	3	3	0.08054	357
43	Bi	Al	3	3	0.07675	213
44	Bi	W	3	3	0.1009	443
45	W	Al	3	3	0.0682	425

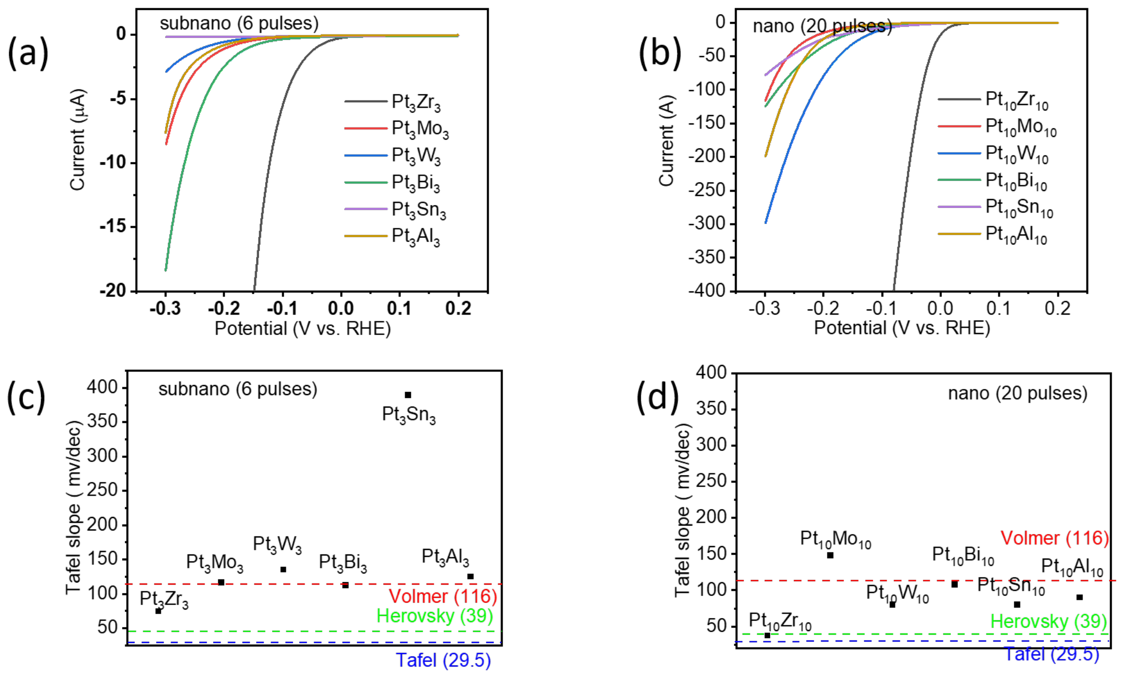

Figure S44. (a, b) LSV recorded in cathodic scans and (c, d) Tafel slope values for Pt-based bimetallic SNPs and NPs in 0.05M H_2SO_4.

Section S6. HSI (HER synergistic effect index)

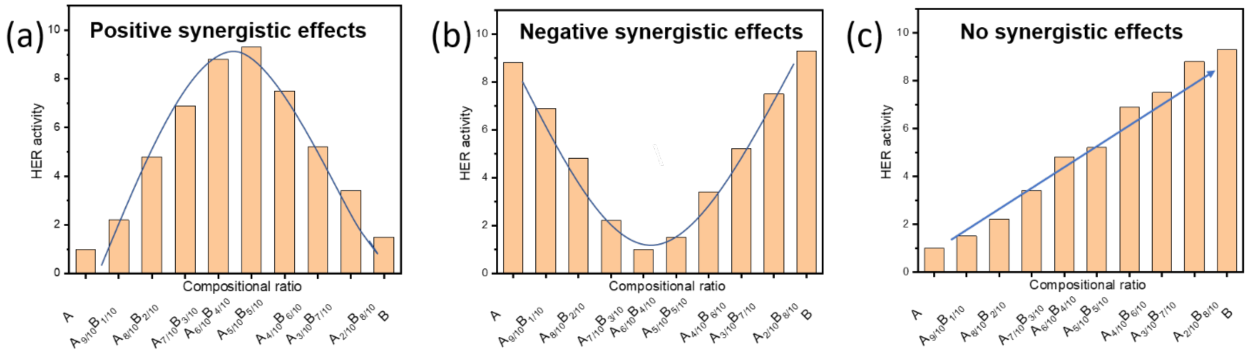

Figure S45. Relationship between HER activity and compositional ratio showing a (a) positive synergistic effect, (b) no synergistic effect, (c) negative synergistic effect.

Three relationships are possible between compositional ratio and HER activity. As shown in Figure S45, they were categorized as positive synergistic effect, negative synergistic effect, or no synergistic effect.[5],[6] To quantify and summarize all possible relationships, the HSI was used.

$$\mathbf{HSI} = (2\boldsymbol{i}_0(\mathbf{M}_x^1\mathbf{M}_y^2) - \boldsymbol{i}_0(\mathbf{M}_x^1) - \boldsymbol{i}_0(\mathbf{M}_y^2))/2\boldsymbol{i}_0(\mathbf{M}_x^1\mathbf{M}_y^2)$$

where $i_0(\mathbf{M}^1)$, $i_0(\mathbf{M}^2)$, and $i_0(\mathbf{M}_x^1\mathbf{M}_y^2)$ represent HER exchange current of single component **M**^{1}, single component **M**^{2}, and bimetallic $M^1{}_xM^2{}_y$ (x and y are concentrations of M^1 and M^2, respectively). Based on the HIS, the synergistic effect between two metals for HER can be determined.

Section S7. Determination of bond length

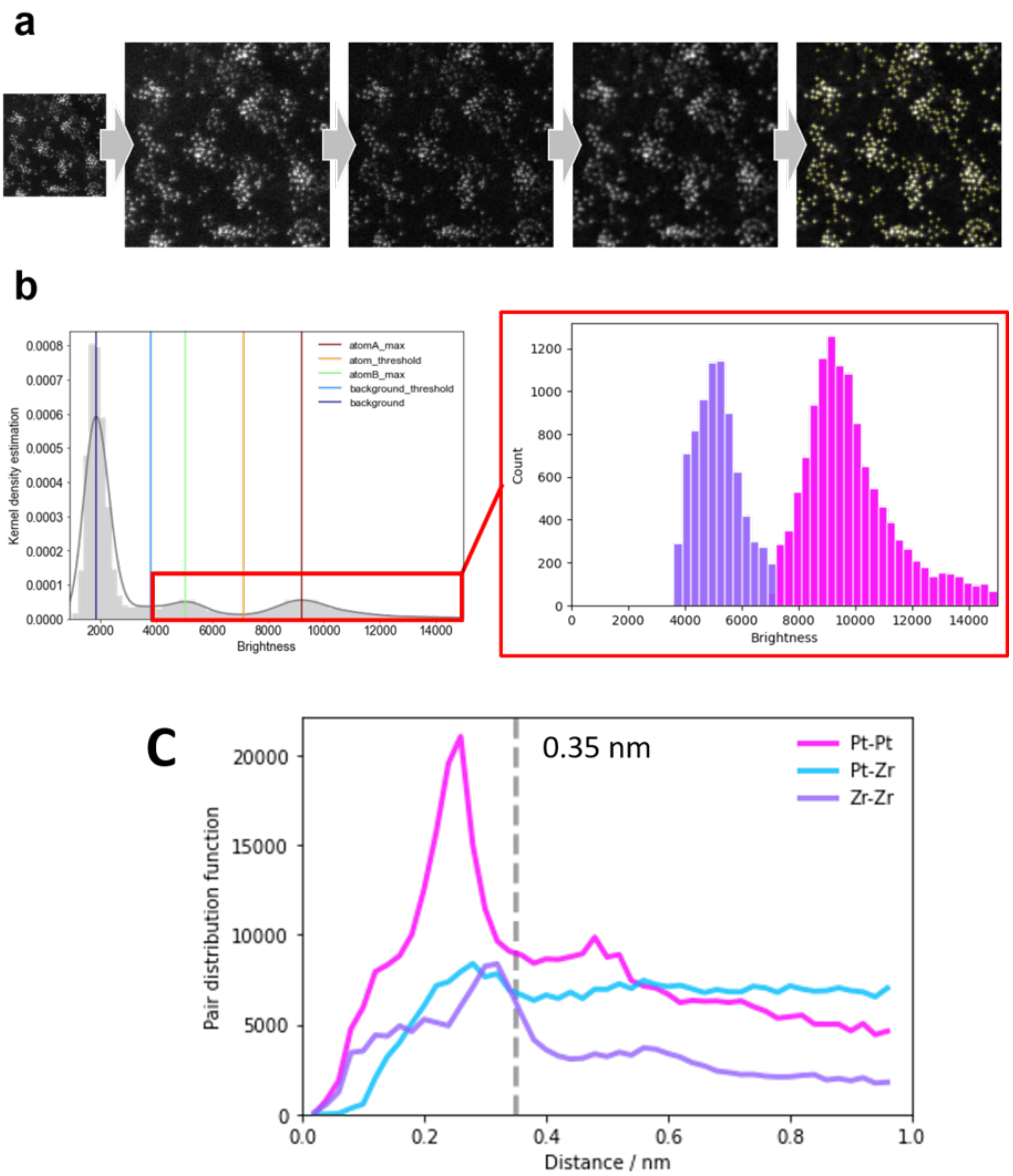

Figure S46. (a) Treatment procedures for ADF-STEM images, (b) subtraction of background and background thresholds for different atoms, (c) pair distribution functions for Pt-Pt, Pt-Zr, and Zr-Zr bonds

Section S8. DFT calculations

To explore the most stable structure metastable structures of Pt_xZr_y SNPs (x + y = 6), the CALYPSO (Crystal structure AnaLYsis by Particle Swarm Optimization) program[7–9] and Gaussian 16[10] program package were used. CALYPSO is a program based on the particle swarm optimization (PSO) algorithm and is used for global optimization. Gaussian 16 is used for local optimization. The structure of each SNP is roughly optimized using the PM7[11] method and then optimized accurately by B3LYP[12–14] functional and lanl2dz [15–17]basis sets. The spin multiplicity considered was in range from singlet to dectet. Then the adsorption structure of a hydrogen atom was optimized on the SNP with the lowest formation energy in each composition ratio. B3LYP/lanl2dz was used for the optimization. All adsorption structures were considered and the most stable adsorption site was explored.

Table S4. Table of the most stable cluster structures and the lowest Eads [kcal mol^{-1}] against composition ratio. The green, gray, and pink spheres indicate Zr, Pt, and H atoms, respectively. The threshold value of bond length between metal atoms is 3 Å

	Optimized SNP structures	Optimized H adsorption structures	The lowest E_{ads}	The most stable adsorption site
Zr_6	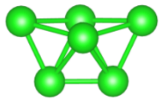 Spin Multiplicity: 1	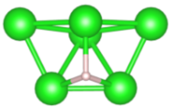 Spin Multiplicity: 2	-24.9	Zr hollow site
Zr_5Pt_1	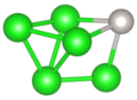 Spin Multiplicity: 1	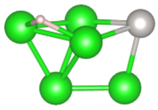 Spin Multiplicity: 2	-26.8	Zr-Zr bridge site
Zr_4Pt_2	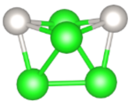 Spin Multiplicity: 1	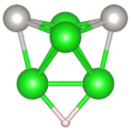 Spin Multiplicity: 2	-19.1	Zr-Zr bridge site
Zr_3Pt_3	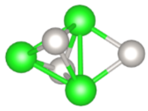 Spin Multiplicity: 1	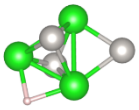 Spin Multiplicity: 2	-15.4	Zr-Zr bridge site
Zr_2Pt_4	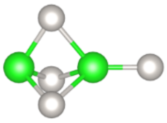 Spin Multiplicity: 1	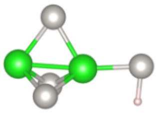 Spin Multiplicity: 2	-1.3	Pt ontop site
Zr_1Pt_5	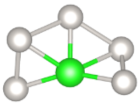 Spin Multiplicity: 3	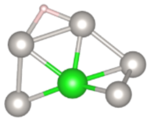 Spin Multiplicity: 2	-14.5	Pt-Pt bridge site
Pt_6	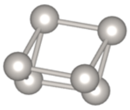 Spin Multiplicity: 7	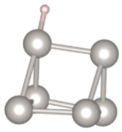 Spin Multiplicity: 6	-13.5	Pt ontop site

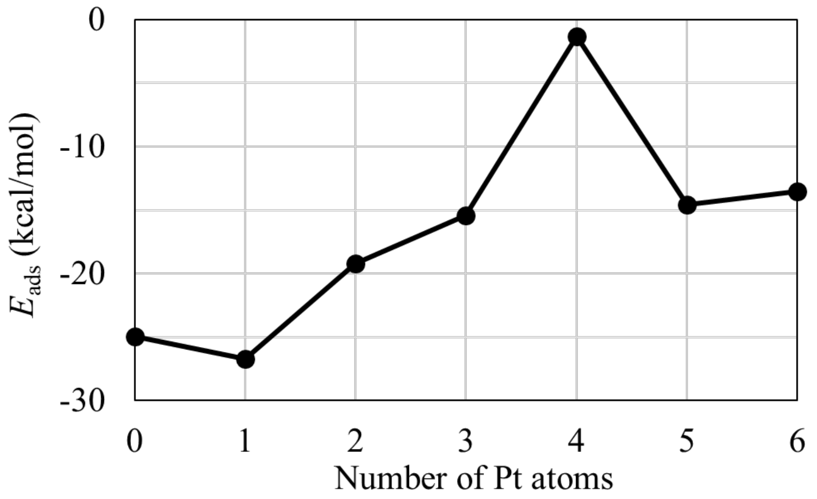

Figure S47. Effect of the number of Pt atoms in the SNP on E_{ads}. In each case, total number of atoms in one SNP was fixed to six, and the number of Zr atoms is given as the difference between six and the number of Pt atoms

References

[1] T. Imaoka, T. Toyonaga, M. Morita, N. Haruta, K. Yamamoto, *Chem. Commun.* **2019**, *55*, 4753–4756.

[2] J. Zhu, L. Hu, P. Zhao, L. Y. S. Lee, K. Y. Wong, *Chem. Rev.* **2020**, *120*, 851–918.

[3] C. A. Schneider, W. S. Rasband, K. W. Eliceiri, *Nat. Methods* **2012**, *9*, 671–675.

[4] J. Schindelin, I. Arganda-Carreras, E. Frise, V. Kaynig, M. Longair, T. Pietzsch, S. Preibisch, C. Rueden, S. Saalfeld, B. Schmid, J. Y. Tinevez, D. J. White, V. Hartenstein, K. Eliceiri, P. Tomancak, A. Cardona, *Nat. Methods* **2012**, *9*, 676–682.

[5] R. Li, J. Chen, T. C. Cesario, X. Wang, J. S. Yuan, P. M. Rentzepis, *Proc. Natl. Acad. Sci. U. S. A.* **2016**, *113*, 13612–13617.

[6] B. M.C., *J. Infect. Dis.* **2015**, *137*, 122–130.

[7] Y. Wang, J. Lv, L. Zhu, Y. Ma, *Phys. Rev. B - Condens. Matter Mater. Phys.* **2010**, *82*, 094116.

[8] Y. Wang, J. Lv, L. Zhu, Y. Ma, *Comput. Phys. Commun.* **2012**, *183*, 2063–2070.

[9] B. Gao, P. Gao, S. Lu, J. Lv, Y. Wang, Y. Ma, *Sci. Bull.* **2019**, *64*, 301–309.

[10] M. J. Frisch, G. W. Trucks, H. B. Schlegel, G. E. Scuseria, M. a. Robb, J. R. Cheeseman, G. Scalmani, V. Barone, G. a. Petersson, H. Nakatsuji, X. Li, M. Caricato, a. V. Marenich, J. Bloino, B. G. Janesko, R. Gomperts, B. Mennucci, H. P. Hratchian, J. V. Ortiz, a. F. Izmaylov, J. L. Sonnenberg, Williams, F. Ding, F. Lipparini, F. Egidi, J. Goings, B. Peng, A. Petrone, T. Henderson, D. Ranasinghe, V. G. Zakrzewski, J. Gao, N. Rega, G. Zheng, W. Liang, M. Hada, M. Ehara, K. Toyota, R. Fukuda, J. Hasegawa, M. Ishida, T. Nakajima, Y. Honda, O. Kitao, H. Nakai, T. Vreven, K. Throssell, J. a. Montgomery Jr., J. E. Peralta, F. Ogliaro, M. J. Bearpark, J. J. Heyd, E. N. Brothers, K. N. Kudin, V. N. Staroverov, T. a. Keith, R. Kobayashi, J. Normand, K. Raghavachari, a. P. Rendell, J. C. Burant, S. S. Iyengar, J. Tomasi, M. Cossi, J. M. Millam, M. Klene, C. Adamo, R. Cammi, J. W. Ochterski, R. L. Martin, K. Morokuma, O. Farkas, J. B. Foresman, D. J. Fox, **2016**, Gaussian 16, Revision C.01, Gaussian, Inc., Wallin.

[11] J. J. P. Stewart, *J. Mol. Model.* **2013**, *19*, 1–32.

[12] A. Becke, *Phys. Rev. A* **1988**, *38*, 3098–3100.

[13] T. Lecklider, *EE Eval. Eng.* **2011**, *50*, 36–39.

[14] A. A. Frost, B. Musulin, *Hydrocarb. J. Chem. Phys.* **1953**, *21*, 5648.

[15] P. J. Hay, W. R. Wadt, *J. Chem. Phys.* **1985**, *82*, 270–283.

[16] W. R. Wadt, P. J. Hay, *J. Chem. Phys.* **1985**, *82*, 284–298.

[17] P. J. Hay, W. R. Wadt, *J. Chem. Phys.* **1985**, *82*, 299–310.

Author Contributions

Most of the experiments involving the preparation of SNPs, electrochemical measurements, and characterizations were conducted by Q.Z. STEM observations and analyses of chemical bond number were conducted by Y.A. The DFT calculations were conducted by T.Y. The manuscript was co-written by Q.Z., A.K., T.I., and K.Y. This study was conceived by T.I. and K.Y.
